# Cognitive dedifferentiation in later life: longitudinal findings from the Lothian Birth Cohort 1936

**DOI:** 10.1093/geronb/gbaf189

**Published:** 2025-10-04

**Authors:** Joanna E Moodie, Janie Corley, Ian J Deary, Simon R Cox

**Affiliations:** Lothian Birth Cohorts, Department of Psychology, The University of Edinburgh, Edinburgh, United Kingdom; Lothian Birth Cohorts, Department of Psychology, The University of Edinburgh, Edinburgh, United Kingdom; Lothian Birth Cohorts, Department of Psychology, The University of Edinburgh, Edinburgh, United Kingdom; Lothian Birth Cohorts, Department of Psychology, The University of Edinburgh, Edinburgh, United Kingdom

**Keywords:** Cognitive aging, Dispersion, General cognitive functioning, Longitudinal cohort

## Abstract

**Objectives:**

In the cognitive aging literature, the dedifferentiation hypothesis refers to cognitive skills becoming more interrelated in older adulthood. Here, we report evidence for cognitive dedifferentiation in the Lothian Birth Cohort 1936 (LBC1936).

**Methods:**

The LBC1936 is a narrow-age cohort assessed at 5 waves between ages 70 and 82. We analyzed data from 418 participants (49% male) who provided cognitive data at all 5 waves.

**Results:**

In single-order structural equation models, the percentage of variance that general cognitive functioning (*g*) accounted for across 13 cognitive tests increases by wave; w1 to w5: 25%, 27%, 29%, 31%, 36%, and the group-level rate of dedifferentiation closely tracked the group-level rate of cognitive decline (*r * =  −.991, *p* = .001). A hierarchical model, which included 4 cognitive domains as mid-level factors, provides evidence of cognitive dedifferentiation at the cognitive domain level: fluid cognitive domains (Visuospatial Skills, Processing Speed, and Verbal Memory) converged, and Crystallised Ability became less influential on the structure of *g* over time. We also show that this group-level measure of dedifferentiation reflects the individual-level measure of dispersion (people tend to score more similarly across different cognitive tests with advancing age), *r  *=  −.989, *p* = .001.

**Discussion:**

The current results have implications for longitudinal *g* modeling choices: it cannot be assumed that *g*’s composition is the same over time. Future longitudinal research will be important in clarifying the incremental validity, determinants, mechanisms, and implications of cognitive differentiation and dedifferentiation across the lifespan.

Cognitive test scores tend to be positively correlated, such that performance level on one cognitive test predicts similar performance on all other tests across a broad range of cognitive domains. When scores from multiple cognitive tests spanning various cognitive domains are considered, a robust component or latent factor of general cognitive functioning (*g*) can be derived, which typically explains a large minority (∼30%–40%) of the variance (Johnson et al., 2004, 2008; Salthouse, 2005). This construct is one of the most replicated phenomena in psychological science (Deary, 2012; Panizzon et al., 2014), and its individual differences correlate with important life outcomes, including everyday functioning, health, illness, aging, dementia, and mortality (Deary et al., 2009; Jonas et al., 2022).

A key debate remains: to what extent does the interrelation between cognitive test scores change over the lifespan? Changes in the covariation among cognitive test scores over time are referred to as cognitive differentiation (where cognitive skills become more distinct from one another) and cognitive dedifferentiation (where cognitive skills become more interrelated). The cognitive differentiation-dedifferentiation hypothesis suggests that differentiation occurs in early life (e.g., Burt, 1954; Li et al., 2004), and dedifferentiation tends to occur in later life (e.g., Babcock et al., 1997; Baltes et al., 1980; Blum & Holling, 2017; Deary et al., 2004; de Frias et al., 2007; Ghisletta & de Ribaupierre, 2005; Ghisletta & Lindenberger, 2003, 2004; Hülür et al., 2015). However, some studies find little or no evidence of such effects, and others find more complex patterns (Anstey et al., 2003; Batterham et al., 2011; Breit et al., 2025; de Mooij et al., 2018; Hartung et al., 2018; Sims et al., 2009; Tucker-Drob, 2009; Tucker-Drob & Salthouse, 2008; Whitley et al., 2016).

One possible explanation for some of any cognitive dedifferentiation in older age is the general decline in cognitive functioning. Previous findings provide evidence that, cross-sectionally, there is increased dedifferentiation with lower cognitive performance (Deary & Pagliari, 1991; Deary et al., 1996; Detterman & Daniel, 1989; Spearman, 1927). On a biological level, there might be age-related declines in neuronal specificity, which could then affect cognitive performance (Cox et al., 2016; Li et al., 2001; Park & Reuter-Lorenz, 2009; Raz, 2000).

Several conceptual models are relevant to the phenomenon of cognitive dedifferentiation. Tucker-Drob et al. (2019) define the concepts of static and dynamic dedifferentiation. Static dedifferentiation refers to the cross-sectional observation that cognitive abilities become more highly correlated in older adults, suggesting convergence across cognitive domains in later life. Dynamic dedifferentiation focuses on intraindividual change over time, suggesting that, as individuals age, domain-based patterns of cognitive change become more synchronized across domains, which, over time, increases the proportion of variance accounted for by *g*. Tucker-Drob et al. (2019) showed that the shared variance in individual differences in cognitive change increased from about 45% at age 35 to about 70% by age 85. Accumulated effects of cognitive changes, across many years, could help to explain static dedifferentiation effects in later life. Another concept closely connected to dedifferentiation is that of dispersion (Lindenberger & Baltes, 1997), which refers to the *intraindividual* variability in performance across different cognitive tasks (in contrast to dedifferentiation, which is, by definition, a group-level construct). Changes in dispersion in older age may reflect dedifferentiation and have similarly been interpreted as a marker of neurological decline or reduced efficiency in neural processing (Buczylowska & Petermann, 2018; Lindenberger & Baltes, 1997; Mella et al., 2016; Rapp et al., 2005).

Understanding cognitive differentiation and dedifferentiation is meaningful because these phenomena help to characterize cognitive trajectories across the life course and could aid the identification of early markers of cognitive decline and distinguish healthy aging from pathological aging. For example, a study found that cognitive dedifferentiation was more pronounced in two samples with marked cognitive decline compared to controls, when controlling for age, sex, and education (Wallert et al., 2021), suggesting that measures of dedifferentiation could provide information of clinical relevance. However, the incremental predictive value of cognitive dedifferentiation in addition to cognitive decline is yet to be clearly demonstrated.

To date, much of the research on cognitive differentiation and dedifferentiation has relied on cross-sectional designs, comparing different individuals at different life stages. While often informative, cross-sectional designs are limited by potential confounding factors, for example, factors that might differ across age groups and cohort effects. Longitudinal designs, in contrast, allow for the measurement of changes in cognitive interrelations over time within the same individuals, enabling direct analysis (e.g., Hülür et al., 2015). Inconsistent results in this field might be due to tests covering different domains, samples with wide age ranges that include periods of differentiation, structural stability, and dedifferentiation (thus masking effects), small and unrepresentative samples, or unaccounted for pathology. Thus, investigations in well-characterized cohorts of exclusively older adults across a wide range of cognitive tests and domains can substantially inform this line of enquiry.

In the current paper, we test for evidence of cognitive dedifferentiation using data from the Lothian Birth Cohort 1936 (LBC1936), a narrow-age longitudinal cohort with data from five waves collected approximately every 3 years between the ages of 70 and 82 years. By analyzing this informative data set, we aim to provide new insights into the dynamics of cognitive dedifferentiation in older adulthood.

## Method

### Participants

The LBC1936 is a longitudinal study of a sample of community-dwelling older adults who were born in 1936, most of whom took part in the Scottish Mental Survey of 1947 when they were ∼11 years old, and who volunteered to participate in this cohort study at ∼70 years old (Deary et al., 2007; ­Taylor et al., 2018; https://lothian-birth-cohorts.ed.ac.uk/). The inclusion criteria for the LBC1936 cohort were having been at school in Scotland for the Moray House Test at age 11, and to have been living in Edinburgh for the start of the cohort testing in 2004. The current sample were all White Scottish, and the data included here were collected in Edinburgh between 2004 and 2019. w1*N *= 418 participants completed at least one cognitive test at all five waves (waves 1 to 5, w1 to w5). These 418 participants constitute the present analytic sample (49% male). Data were collected approximately every 3 years. The mean age (years) per wave was as follows: w1 = 69.46 (*SD* = .85), w2 = 72.44 (*SD* = .72), w3 = 76.20 (*SD* = .68), w4 = 79.28 (*SD* = .63), and w5 = 82.00 (*SD* = .48). The minimum number of participants for any one test at any wave was *N = *371 (89% of the sample), for both inspection time total in w4 and verbal paired associates in w5 (a grid showing the completeness of data by test is shown in Supplementary Figure 1 [see online supplementary material]).

There was an association between w1 cognitive test scores and participants having any missing waves from w2 to w5 (0 = complete data, 1 = some missing data on the relevant ­cognitive test w2 to w5), such that people with missing data had slightly lower cognitive scores at w1 (across tests, mean β = −.132, *SD* = .106, mean *p* = .0002, *SD* = .0007, the full results are in Supplementary Table 1 [see online supplementary material]). Therefore, the subset of participants with complete data (*N* = 418) that we include in this analysis tended to have slightly higher cognitive scores than the full sample. Further differences between completers and non-completers in the LBC1936 cohort were analyzed in a previous study (Corley et al., 2023).

To assess whether results reflected normative aging processes, we conducted sensitivity analyses excluding those with a subsequent dementia diagnosis. No participants had dementia at w1. Dementia diagnoses for the LBC1936 are ascertained through a three-step process: Electronic Health Records and death certificate data, a home visit to some participants, and a consensus review board meeting with experienced dementia experts (Mullin et al., 2023). The LBC1936 is an ongoing longitudinal study, and the dementia diagnoses included here were correct to the best of our knowledge as of the 6th of March 2025. Out of the *N = *418 participants included in the current study, *N = *58 have had subsequent dementia diagnoses. Therefore, *N *= 360 were included in our sensitivity analyses.

The LBC1936 study was given ethical approval by the Multi-Centre Research Ethics Committee for Scotland (MREC/01/0/56), the Lothian Research Ethics Committee (LREC/2003/2/29), and the Scotland A Research Ethics Committee (07/MRE00/58). All participants gave written informed consent.

#### Cognitive tests

The 13 cognitive tests are described in detail elsewhere (Deary et al., 2007; Ritchie et al., 2016; Tucker-Drob et al., 2014). They cover four cognitive domains:

- Visuospatial Skills—Block design (Wechsler, 1997a), Matrix reasoning (Wechsler, 1997a), and Spatial span (Wechsler, 1997b)
- Processing Speed—Symbol search (Wechsler, 1997a), Digit-symbol substitution (Wechsler, 1997a), Inspection time (Deary et al., 2004), and Four-choice reaction time (Deary et al., 2001)
- Verbal Memory—Verbal paired associates (Wechsler, 1997b), Logical memory (Wechsler, 1997b), and Digit span backwards (Wechsler, 1997a)
- Crystallised Ability—Verbal fluency (Lezak et al., 2004), National Adult Reading Test (NART; Nelson & Wilson, 1991), Weschler Test of Adult Reading (WTAR; Wechsler, 2001)

See Supplementary Tables 2 and 3 (see online supplementary material) for details of individual cognitive tests. Two cognitive tests had skewed distributions and so were transformed: inspection time total was squared, and choice reaction time was multiplied by the power of minus two. All variables were scaled before being entered into the structural equation analysis (SEM) models. Correlations between the 13 cognitive tests for each wave are shown in Supplementary Figure 2 (see online supplementary material).

#### Latent *g* models

We used SEM with the lavaan package (v 0.6.17, Rosseel, 2012) in R (v4. 2.0, R Team, 2022) to model a latent *g* factor at each wave. We ran two main types of models to assess and characterize any dedifferentiation effects: single-order models and a hierarchical model.

**Figure 1.**
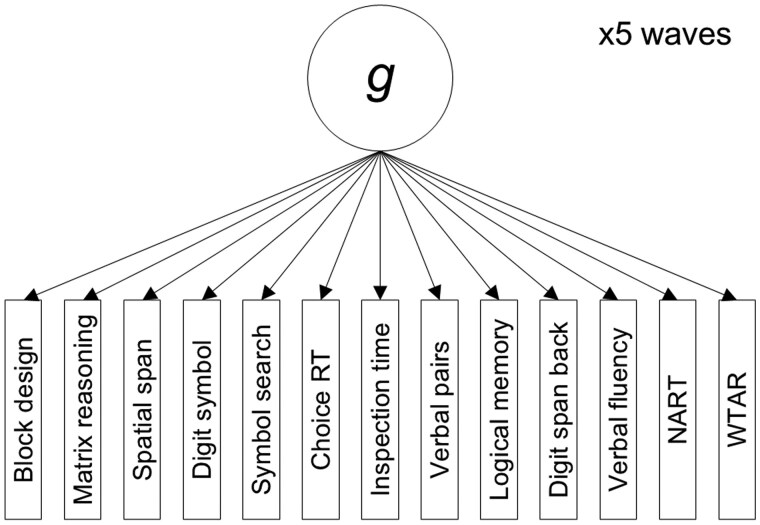
Diagram of single-order latent *g* model structure. RT = Reaction Time; NART = National Test of Adult Reading; WTAR = Wechsler Test of Adult Reading.

For the single-order models, the 13 cognitive tests from each wave directly loaded onto the relevant wave’s *g* factor (the 13 cognitive tests at w1 were loaded onto *g*_w1, those from w2 onto *g_*w2, etc.). Figure 1 shows a diagram of the single-order models. We ran four types of single-order models:

- Model A: *g* intercept estimated, cognitive test intercepts fixed to 0:
  - Model A (all waves; this is the main single-order model of interest)
  - Model A1, A2, A3, A4, A5 (separate waves, 5 models)
- Model B: *g* intercept fixed to 0, cognitive test intercepts estimated:
  - Model B (all waves)
  - Model B1, B2, B3, B4, B5 (separate waves, 5 models)

Supplementary Figure 5 (see online supplementary material) shows an illustration showing the different model specifications in Model A and Model B. Model A (all waves) was the main model of interest, because in order to get relative *g* scores across waves (which enabled the testing of correlations between group-level dedifferentiation effects and group-level cognitive change), it was necessary to estimate the latent intercept and fix cognitive test intercepts to zero, and in order to run the relevant tests (e.g., tests of measurement invariance), it was also necessary to include all waves in the same model. Usually, to estimate latent scores within SEM, the latent intercept is fixed to zero, and cognitive test intercepts are estimated (as in Model B, and we include Model B to validate our results with this more conventional latent variable modeling approach). Supplementary Figure 6 (see online supplementary material) illustrates that the means of the *g* estimates from Model A change over time (reflecting that, generally, older adults do not score as highly as their younger selves), whereas in Model B, the group-level *g* estimates at each wave have a mean ≈ 0. For all-waves models, we set covariances between *g*s at different waves to zero because our aim was to calculate wave-specific latent *g* scores. We also ran *g* measurement models for each of the waves separately (models numbered 1 to 5, denoting each of the five waves) for validation and comparison purposes, and the lavaan code and results for all models are presented in the Supplementary material.

In these single-order models, we included a covariance between NART and WTAR, which have particularly high within-wave correlations in all waves (at each wave, *r *> .794), as these are highly similar cognitive tests involving word pronunciation.

**Figure 2.**
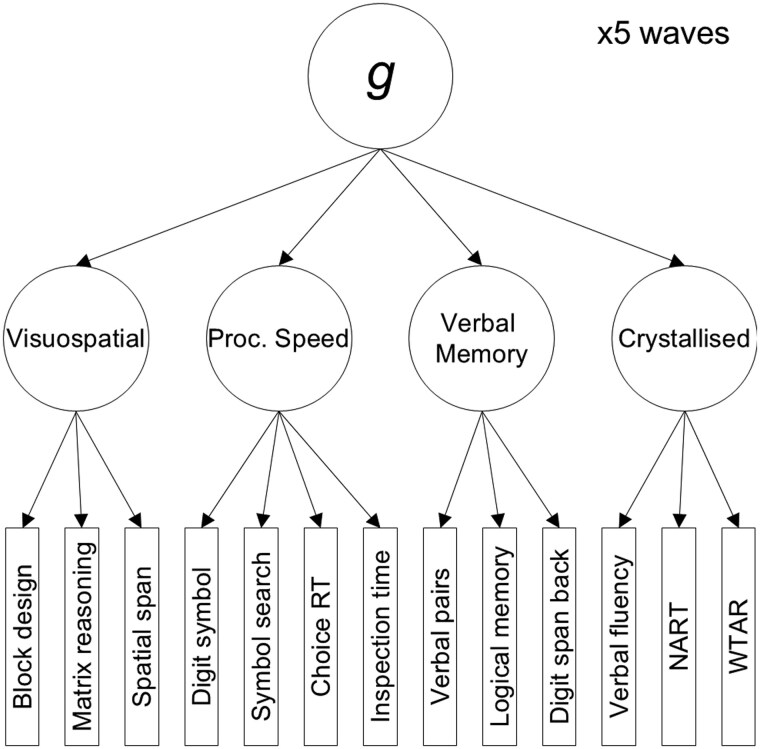
Diagram of the hierarchical latent *g* model structure. Proc. Speed = Processing Speed; RT = Reaction Time; NART = National Adult Reading Test; WTAR = Wechsler Test of Adult Reading.

An additional hierarchical analysis was conducted where, for each wave, the 13 cognitive tests load onto four cognitive domains: Crystallised Ability, Visuospatial Skills, Verbal Memory, and Processing Speed, and the four domains load onto *g* (see Figure 2), as modeled previously in this cohort (Ritchie et al., 2016; Tucker-Drob et al., 2014). We used a typical structural equation modeling framework and fixed covariances between *g* factors from different waves to 0. This modeling framework is comparable to Model B listed above, as the one used in Model A (fixing cognitive test loadings to 0) can distort mid-level factor loadings in hierarchical SEM models, which are the loadings of interest in this analysis. We did not specify any test-wise covariances in the hierarchical model, as our aim was for the domain factors to capture the maximum shared variance among their respective indicators.

#### Data availability

To access the Lothian Birth Cohort data, see https://lothian-birth-cohorts.ed.ac.uk/data-access-collaboration. The code for all structural equation models is included in the Supplementary material. R version 4.2.0 (R Team, 2022) and lavaan 0.6.17 (Rosseel, 2012) were used for analyses.

## Results

Descriptive statistics of all cognitive tests for all waves are in Supplementary Table 3 (see online supplementary material). Their distributions are shown in Supplementary Figure 3 (see online supplementary material), and their mean changes across waves in Supplementary Figure 4 (see online supplementary ­material). The standardized loadings and related results from the single-order latent variable models are shown in Supplementary Tables 5–8 (see online supplementary material), and those from the hierarchical latent variable model are in Supplementary Table 9 (see online supplementary material).

### Single-order models (without domains)

In this section, unless stated otherwise, we report the results from Model A (all waves), but results from all models are in the Supplementary material.

The model fits for models A, A1–A5, B, and B1–B5 are ­presented in Supplementary Table 4 (see online supplementary ­material). Common quality of model fit criteria of CFI > .95, TLI > .95, RMSEA < .06, and SRMR < .08 (Hu & Bentler, 1999) are of less relevance than usual, due to our modeling choices. As expected, the model fit of the “all waves” models was poorer than for those that model each wave separately. This is because the longitudinal covariance paths that we would expect to be high across *g* scores at different waves were intentionally not included in the “all waves” models, because our aim was to calculate wave-specific latent *g* scores. However, their strong correspondence to our separate per-wave measurement models (discussed in the next section) indicates no model misspecification. Additionally, the fits of the separate-wave models are somewhat poorer than classic criteria for model fit. This is because, for reasons described above in the methods section, within-domain covariances were not included in these single-order models.

#### Correlations between g estimates across waves

There are strong correlations between *g* estimates at each wave (Model A, all waves: *r* range = .79 to .93). These correlations decrease in magnitude as the time between waves increases. For example, w1 *g* scores correlate at *r = *.89 with w2, *r = *.87 with w3, *r = *.86 with w4, and *r = *.79 with w5. In other words, people score similarly across waves (e.g., people who score highly compared to the rest of the group at one wave are likely to score highly at other waves), although this relationship weakens slightly with increased time between testing sessions. A similar trend was found for Model B (all waves, see Supplementary Figure 6 [see online supplementary material]), consolidating this finding.

#### Outcome reliability between models

To test the impact of different model types on outputs, we tested the consistency of *g* scores between the model types, and the results suggest high reliability. First, we tested the correlations of extracted *g* scores between “all waves” models versus the separate waves models. All correlations for extracted *g* scores matched by waves were, for Model A (between all waves and separate models), *r *> .995, and all factor congruence for loadings > .98; and for all correlations for Model B (between all waves and separate models), *r = *1, and all factor congruence for loadings > .97. Then, we tested the correlations between the extracted *g* scores for Model A (all waves) and Model B (all waves). The extracted *g* estimates from Model A and Model B (all waves) were highly correlated, at *r *> .983 for all five waves (all measures of factor congruence for loadings > .97). Therefore, whereas the model syntax and model fits are different between the model types, the outcomes in terms of *g* estimates and loadings have high reliability.

#### Lack of weak measurement invariance: loadings of tests on g are not stable across waves

Having established the validity and reliability of our measurement models, we began to examine potential dedifferentiation effects. We first conducted a formal test of measurement invariance to test whether the 13 cognitive tests have similar loadings on the *g* factor across the waves (testing weak measurement invariance). We compared the standard model to one where loadings were fixed for each test across waves. There was no evidence of weak measurement invariance. The results were significant for both Model A (all waves), *p *< 2.2e^−16^, and Model B (all waves), *p* = .0007 (see Table 1 for details), providing evidence that there are differences in how the cognitive tests load onto *g* between waves. However, across the waves, the factor congruence = 1 for Model A (all waves) and > .97 for Model B (all waves), suggesting that the rank-order of test loadings on *g* is well-matched between waves.

**Table 1.** Results from the chi-squared difference test, testing weak measurement invariance (standard model vs model with fixed loadings for each test across waves).

Model type	Model specification	*df*	AIC	BIC	χ^2^	χ^2^ diff.	RMSEA	*df* diff.	*p*
**Model A (all waves)**	Standard	2,070	−7,754	−7,189	21,224				
	Fixed loadings	2,218	−7,331	−6,960	21,742	518	.153	48	<2.2e^−16^
**Model B (all waves)**	Standard	2,010	−8,810	−8,002	20,048				
	Fixed loadings	2,058	−8,820	−8,207	20,133	85	.043	48	.0007

*Note*. These tests were significant, suggesting that there is no weak measurement invariance across waves. df = degrees of freedom; AIC = Akaike information criterion; BIC = Bayesian Information Criterion; diff = difference; RMSEA = Root Mean Square Error of Approximation.

#### The % of variance accounted for by g increases with age

**Figure 3.**
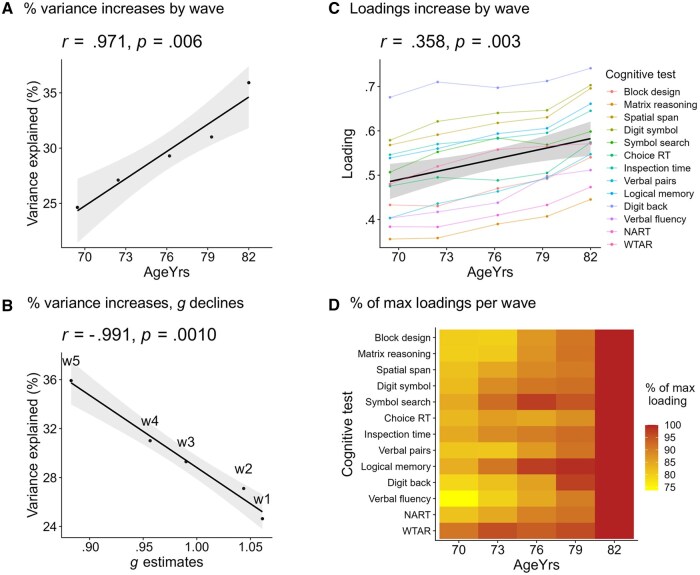
Results from the single-order model of cognitive dedifferentiation in LBC1936. (A) The % variance accounted for by latent *g* increases by wave. (B) Strong negative correlation between % of variance explained and *g* estimates across waves—as % variance increases across waves, *g* estimates decline. (C) Loadings on *g* tend to increase by wave. The black line represents the mean loading for each wave, plotted with the “lm” function. Equivalent figures, for the four different model types presented together, are available in Supplementary Figures 6 and 7 (see online supplementary material). (D) Heatmap of % of max loadings per cognitive test per wave, based on the same data as part (C), shows again that, across tests, loadings tend to increase between w1 and w5. RT = Reaction Time; NART = National Adult Reading Test; WTAR = Wechsler Test of Adult Reading.

Consistent with the dedifferentiation hypothesis of cognitive aging, the variance accounted for by *g* and the *g* loadings increased by wave in all models (see Supplementary Figures 6 and 7 [see online supplementary material]). In Model A (all waves), the variance accounted for was 24.64% at w1, 27.11% at w2, 29.30% at w3, 31.01% at w4, and 35.92% at w5 (correlation with mean age per wave: *r = *.971, *p* = .006, see Figure 3A). The results for the other models are highly similar and are shown in Supplementary Figure 8 (see online supplementary material). This provides direct evidence for dedifferentiation: the amount of variance that the 13 cognitive tests have in common increased over time.

Accordingly, the mean loadings increased over waves (Model A, all waves, w1 to w5 = .488, .511, .533, .550, and .593, see Figure 3C and D). The comparable results for all models are in Supplementary Figure 7 and Supplementary Tables 5–8 (see online supplementary material).

#### Correlations between % variance explained and mean *g* estimates

The % of the variance explained is strongly negatively associated with the group-level mean of individuals’ *g* score estimates, *r = *−.991, *p* = .0010 (see Figure 3B). The group level means (*SDs*) for *g* score estimates were 1.06 (.11), 1.04 (.11), .99 (.11), .96 (.12), and .88 (.13) for w1 to w5, respectively. Therefore, the rate of group-level dedifferentiation appears to closely track the group-level rate of cognitive decline. Note that the dedifferentiation effect does not directly numerically depend on a decrease in *g* within the model, as it also occurred in Model B, where the mean of *g* at each wave **≈** 0 (see above and Supplementary Figures 6 and 7 [see online supplementary material]).

#### Sensitivity analyses: excluding participants with subsequent dementia

We ran an additional analysis to test whether the dedifferentiation effect held when excluding people who received a subsequent dementia diagnosis from the analysis (no participants had confirmed dementia at w1). We ran Model A (all waves) with the *N = *360 participants without a current dementia diagnosis (see results in Supplementary Figure 9 [see online supplementary material]).

As was the case for the full sample, the variance explained increased across the five waves: 24.47%, 26.35%, 27.58%, 27.84%, and 31.58%, although the percentage values were qualitatively lower than for the full sample (full sample w1 to w5: 24.64%, 27.11%, 29.30%, 31.01%, and 35.92%, ­correlation with mean age per wave: *r = *.939, *p* = .018). The correlation between loadings and the mean age per wave was accordingly lower than for the full sample: *r = *.221, *p* = .078 (compared to *r = *.358, *p* = .003).

To test the statistical significance of these reduced effects, we conducted a linear regression model, which showed that there was no significant interaction between sample type (full sample = 0, no dementia sample = 1) and wave (mean age per wave) when predicting loadings (standardized β = −.141, *p* = .406), and also no significant main effect of sample on loadings (standardized β  =  .002, *p* = .988). These results suggest that the sample composition (including people with dementia or not including them) did not significantly affect the results. Future research with a larger sample of participants who develop dementia is required to determine whether the (nonsignificant) reduction in the effect signals an acceleration of dedifferentiation in people who go on to develop dementia.

#### Dispersion and its relation to cognitive dedifferentiation

We also include an analysis of intraindividual dispersion to explore whether dedifferentiation (a group-level statistical phenomenon) is accompanied by changes in dispersion (a measure of the uniformity of cognitive test scores at the individual level). We first assessed whether levels of intraindividual dispersion changed across waves (that is, whether individuals became more or less variable in their performance across cognitive tests over time). To measure group-level dispersion at each wave, we first scaled each variable and then calculated the group-level mean of the individual-level *SD*s across the 13 cognitive tests for each wave. Group-level dispersion decreased over time, showing that individuals’ cognitive profiles became more homogenous with age (Ms (SDs) from w1 to w5: .819 (.204), .806 (.187), .798 (.184), .788 (.192), .750 (.186). The correlation between group-level dispersion and the proportion of variance explained by *g* from Model A (the dedifferentiation effect) was negative and very strong, *r = *−.989, *p* = .001. Analyses restricted to participants without missing data (*N *= 288) gave identical results to 3 decimal places (*r *= −.989, *p* = .001). Additionally, the correlation between group-level mean dispersion and group-level mean *g* scores for each wave was strong and positive, *r = *.955, *p* = .011, showing that cognitive profiles became flatter as *g* scores declined. These results indicate that here, the observed group-level dedifferentiation phenomenon strongly reflects the tendency for individuals’ test score performance across cognitive tests to become more similar with advancing age in later life.

### Hierarchical model (including domains)

We conducted a hierarchical analysis to help clarify whether dedifferentiation effects from single-order models reflect age-related increases in the reliability of individual cognitive tests (as might be indicated by increased domain-to-test ­loadings) and/or whether they are better explained by convergence across cognitive domains (as indicated by increased *g*-to-domain loadings). The latter would strengthen the interpretation of a valid cognitive dedifferentiation effect. The model fit for the hierarchical model is in Supplementary Table 4 (see online supplementary material), and it fits similarly to Model B above (as in Model B, the model fit is poor by design, because covariances between *g* factors at different waves are set to 0).

#### Domain-to-test results

The proportion of variances accounted for from domains-to-tests generally increased over time, 45%, 47%, 48%, 49%, 54% from w1 to w5 (correlation with mean age per wave: *r = *.904, *p* = .035; see change in proportion of variance for each domain in Supplementary Figure 10 and Supplementary Table 10 [see online supplementary material]). However, despite the general increase in proportion of variance explained, we did not find evidence that individual test loadings change significantly with wave (no *FDR Q* values were < .05, see Supplementary Figure 10, panel D [see online supplementary material]) and, across all 13 cognitive tests, there was not a clear pattern of change in loadings over time (correlation with mean age per wave: *r = *.147, *p* = .242, see Supplementary Figure 10, panels B and C [see online supplementary material]). We further established that there was not a significant change in test-based residual variances across waves for individual cognitive tests (no *FDR Q* values < .05 for each of the 13 tests, or across all 13 cognitive tests, *r = *−.127, *p* = .312, see Supplementary Figure 11 [see online supplementary material]). Therefore, we concluded that increased test reliability cannot fully account for the dedifferentiation effect observed in the single-order models.

#### *g*-to-domain results

**Figure 4.**
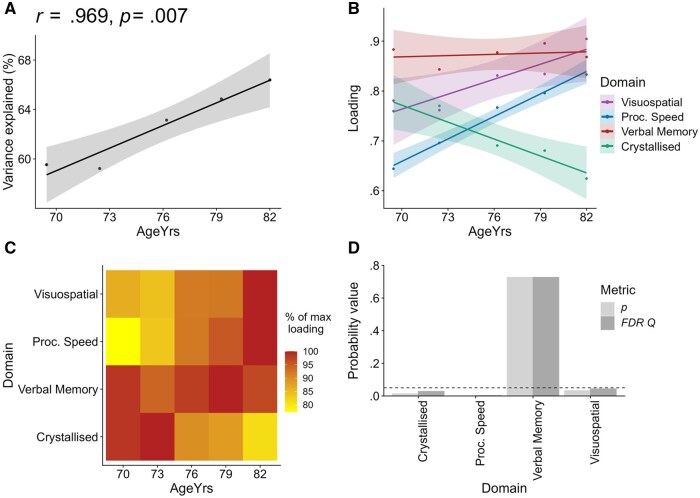
*g*-to-domain results. (A) shows the proportion of variance explained from *g*-to-domains across waves, (B) shows the *g*-to-domain loadings across the five waves, (C) is a heat map showing the same data as in (B) for ease of comparison, and (D) is a bar chart showing that loading magnitudes at the *g*-to-domain level changed significantly (*FDR Q* < .05) across waves for Visuospatial, Processing Speed, and Crystallised Ability domains, and did not change for Verbal Memory. Proc. Speed = Processing Speed; *FDR Q* = False Discovery Rate* Q*.

We then tested whether cognitive dedifferentiation is occurring at the *g*-to-domain level. The results show a general increase in the proportion of variances accounted for over time, 60%, 59%, 63%, 65%, 66% (correlation with mean age per wave: *r = *.969, *p* = .007, see Figure 4A). Further investigations into the cross-wave *g-*to-domain loadings provide insights into cognitive dedifferentiation. When combined across all four domains, there was not a significant general increase in loadings across waves: *r = *.199, *p* = .401. However, at the domain-specific level, there were significant increases in *g-*to-domain loadings for the Visuospatial domain (*r *= 0.908, *p* = .033, *FDR Q* = .044) and the Speed domain (*r* = .993, *p* = .0006, *FDR Q =* .002), which is evidence of cognitive dedifferentiation effects in these fluid domains (see Figure 4B–D).

In contrast, Crystallised Ability showed decreasing loadings on *g* over time (*r* = –.948, *p* = .014, *FDR Q* = .028). This domain remained equally well-defined across waves, as indicated by its relatively stable domain-to-test loadings (*p* = .389; see Supplementary Figure 10, part A), suggesting that its observed decrease in *g-*loading across waves is not attributable to a decline in construct or test quality. Rather, it appears that Crystallised Ability retains domain specificity and that the composition of *g* shifts away from crystallised skills and toward fluid skills in later life. A comparable decrease in loadings over time for Crystallised Ability tests can be seen in the single-order model equivalent, Model B, as NART and WTAR loadings both decrease over time (correlation with mean age per wave: *r = *−.924, *p* = .025, *r = *−.939, *p* = .018, respectively). The verbal fluency test, which loads more weakly onto Crystallised Ability than the other two indicators, does not show a significant increase over time in Model B (*r *= .311, *p* = .610). Therefore, domain-level shifts in *g’*s composition, away from Crystallised Ability, can also be detected in the single-order models.

**Figure 5.**
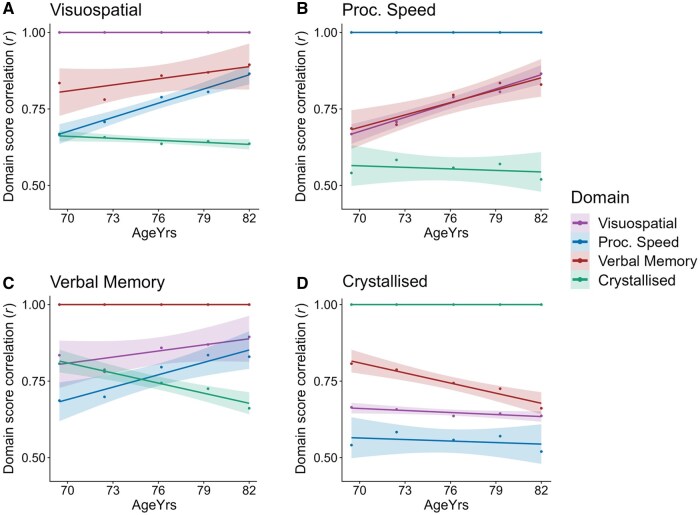
Interdomain correlations for (A) Visuospatial Ability, (B) Crystallised Ability, (C), Verbal Memory, and (D) Processing Speed. The interdomain correlation results relating to this figure are in Supplementary Tables 11 and 12 (see online supplementary material). Proc. Speed = Processing Speed.

For Verbal Memory, there was not a significant change in its *g*-to-domain loading (*r *= .216*, p* = .727, *FDR Q* = .727, see Figure 4B–D). This could be due to its already strong loading onto *g* at baseline (loading = .883; compared to .644 for Speed, 0.760 for Crystallised Ability, and .781 for Visuospatial Skills, all at w1). The strong association between *g* and Verbal Memory could reflect the fact that the tests in this domain are linked to both crystallised and fluid cognitive processes (see Figure 5 and Supplementary Table 12 for interdomain correlations [see online supplementary material]). Perhaps because of a somewhat mixed composition, Verbal Memory already shares a high degree of variance with *g*, and there is less scope for an observable dedifferentiation effect at the level of *g*-to-domain factor loadings.

#### Interdomain correlation results

However, dedifferentiation effects can also be examined at the interdomain level (which could be conceptualized as horizontal effects, compared to *g*-to-domain effects, which are hierarchical). We calculated correlations between extracted domain scores per wave and tested how these interdomain correlations change over time (see Supplementary Table 11 [see online supplementary material]). Results indicate that Verbal Memory becomes increasingly correlated with fluid domains (Processing Speed and Visuospatial Ability) across waves (*r *= .951, *p* = .013, *FDR Q* = .026 and *r = *.778, *p* = .122, *FDR Q* = .0147, respectively; and together, *r = *.905, *p* = .035; see Figure 5C and Supplementary Table 11 [see online supplementary material]), showing that dedifferentiation is occurring between fluid domains. In contrast, Verbal Memory shows a strong and negative correlation with Crystallised Ability across waves (*r *= −.974, *p* = .005, *FDR Q* = .015), reflecting a shift in its alignment away from its crystallised elements and toward its fluid components, as *g* shifts toward fluid domains (see Figure 5C). The interdomain correlations show that, despite Verbal Memory’s loadings onto *g* remaining fairly stable, its broader integration with fluid domains increases in later life, and its correlation with Crystallised Ability decreases, consistent with the cognitive dedifferentiation effects observed at the *g*-to-domain level.

Overall, the results of the hierarchical analysis suggest that dedifferentiation effects affect the composition of *g*, as fluid cognitive domains converge and Crystallised Ability becomes less central to the structure of *g* in later life.

## Discussion

This longitudinal study provides evidence of aging-related group-level cognitive dedifferentiation from ages 70 to 82 years old. Single-level models showed increasing proportions of variance explained and loadings across a multidomain battery of 13 cognitive tests measured on five occasions. Evidence for dedifferentiation was supported by a lack of weak measurement invariance in the measurement of *g* across waves. We also found that the group-level rate of dedifferentiation (measured by % of variance explained and mean loadings per wave) was strongly negatively correlated with the group-level mean of individuals’ *g* estimates. In other words, the group-level rate of dedifferentiation appeared to closely track the group-level rate of cognitive decline. This finding is in line with previous work showing evidence of increased dedifferentiation with lower (cross-sectional) mean levels of cognitive performance (Deary et al., 1996; Detterman & Daniel, 1989; Deary & Pagliari, 1991). However, it is difficult to disentangle here whether dedifferentiation effects are better explained by lower cognitive performance or by age itself (Deary et al., 2004; Tucker-Drob et al., 2019).

The dedifferentiation effect was slightly reduced (although this was not statistically significant) when analyses were conducted on a subset of the sample who have not gone on to receive a dementia diagnosis, pointing to the possibility that while dedifferentiation is a hallmark of normative cognitive aging (as also concluded by Tucker-Drob et al., 2019), the rate of dedifferentiation may accelerate with pathology such as dementia, as found in some other studies (e.g., Wallert et al., 2021). Again, the question of whether such effects are wholly attributable to cognitive decline or involve additional mechanisms requires careful testing and will inform whether cognitive dedifferentiation can serve as an early marker of pathological cognitive decline, beyond traditional assessments of cognitive performance (i.e., individual test scores).

We also observed a decrease in intraindividual dispersion across 13 cognitive tests over time, indicating that individuals’ cognitive profiles became more homogeneous over time in later life. This finding is consistent with some previous findings (Buczylowska & Petermann, 2018; Lindenberger & Baltes, 1997; Mella et al., 2016). This decrease in dispersion occurred alongside the increased proportion of variance explained by *g* and a decrease in cognitive scores. In other words, the cognitive dedifferentiation and cognitive decline effects we found here are mirrored in the intraindividual cognitive scores becoming increasingly interdependent with age. As people differ in the amount of dispersion they exhibit, dispersion offers an attractive tool to better understand the phenomenon of cognitive dedifferentiation (e.g., its determinants, neural correlates, and functional outcomes).

Crucial to our interpretation of the dedifferentiation effects, a hierarchical model showed that the proportion of variances tended to increase across waves at both the domain-to-test and *g*-to-domain levels, but the domain-to-test level loadings did not generally increase. At the *g-*to-domain level, loadings increased for Visuospatial Skills and Processing Speed, decreased for Crystallised Ability, and remained stable for Verbal Memory. Interdomain correlations showed that Verbal Memory exhibits convergence with the other fluid domains (Visuospatial Skills and Processing Speed), suggesting that although there were no dedifferentiation effects evident at the *g*-to-domain level (perhaps due to high baseline correlations with *g*), it still became more strongly correlated with other fluid domains over time. Concurrently, Crystallised Ability may maintain or even increase its independence as other domains converge into a more fluid-driven general factor. These findings have implications for longitudinal *g* modeling decisions—it cannot be assumed that the *g* factor’s composition is the same over time.

The current results contrast with the findings of Tucker-Drob et al. (2019), who found no evidence of static dedifferentiation in their meta-analysis across 22 longitudinal data sets, with mean baseline ages ranging from 35 to 85 years old and between two and 17 cognitive outcomes per data set. Whereas their analyses aggregated across multiple heterogeneous cohorts, our findings are derived from a single narrow-age cohort, potentially allowing for more consistent aging-related patterns to emerge. The results we find might be explained by dynamic dedifferentiation, a term coined by Tucker-Drob et al. (2019). Whilst the current paper did not conduct a direct test of dynamic dedifferentiation, that is, it did not test whether correlations in cognitive change increased with age (given the narrow age range of the LBC1936, and only five available waves of data, which limits the ability to have at least three instances for >1 slope parameter), we have previously reported that longitudinal cognitive changes in the LBC1936 show ­correlated patterns across domains (Corley et al., 2023; Tucker-Drob et al., 2014). It is possible that the dedifferentiation effects observed in our data (“static dedifferentiation”) reflect, in part, the cumulative effects of aging-related declines becoming more interrelated over time (“dynamic dedifferentiation”). The fact that cognitive domains are declining in tandem and are increasingly sharing variance points to the potential presence of common underlying causes as an Occam’s razor explanation (see, e.g., Baltes & Lindenberger, 1997).

The LBC1936 cohort is well-placed to characterize dedifferentiation between 70 and 82 years old, with a rich 13-test cognitive test battery and five waves of data spanning ∼12 years. This data set allowed us to observe within-individual changes over time, a meaningful advantage over cross-sectional studies, which are limited by potential confounding factors such as cohort effects. Cross-sectional data can provide some insights into aging, but, unlike longitudinal data, it cannot capture the complexity and dynamic within-person phenomenon of cognitive aging (Fjell & Walhovd, 2012; Raz & Lindenberger, 2011; Salthouse, 2011). Future longitudinal research covering different stages of the human lifespan will improve understanding of the developmental trajectory of differentiation and dedifferentiation effects, and research directly involving neurobiological properties could identify key mechanisms behind these phenomena.

It should be noted that a prevalent limitation of longitudinal studies is participant drop-out, particularly when systematic. There was some evidence of systematic drop-out here, as people who had slightly lower cognitive test scores on w1 were more likely to have missing data between w2 and w5 than those with cognitive data at all five waves, and we have previously characterized other ways in which this cohort—like many others—suffers from healthy selection bias (Corley et al., 2023). We only included data from people who had cognitive data at all five waves in the current sample, so our characterization of dedifferentiation is based on a subsample of people with slightly higher cognitive test scores than the full sample, which could have unknown effects on, for example, the reported rate of dedifferentiation. Future research is required to establish links between baseline cognitive performance levels and the rate of dedifferentiation in later life.

Due to the inclusion criteria of the LBC1936 cohort, all participants in this study are White Scottish. Future research incorporating more racially and ethnically diverse samples would be valuable in assessing the generalizability of dedifferentiation effects in older age.

Future research aimed at examining and understanding the day-to-day effects of cognitive dedifferentiation, in addition to cognitive decline, could prove beneficial for developing targeted interventions and strategies to support cognitive functioning and daily life in aging populations.

## Conclusion

Overall, this study provides longitudinal evidence of cognitive dedifferentiation between ages 70 and 82, supporting the hypothesis that cognitive skills become increasingly interrelated in later life. We show that the group-level rate of this effect is closely linked to the observed rate of group-level cognitive decline. Over time, the composition of the general factor of cognitive functioning (*g*) shifts: fluid cognitive abilities become increasingly central to *g*, while crystallised abilities appear to contribute less. These findings challenge the assumption that the structure of *g* remains stable with age, and therefore, they also have important implications for how *g* is modeled across the lifespan. Future longitudinal research will be key in clarifying the incremental validity, determinants, mechanisms, and implications of cognitive differentiation and dedifferentiation across the lifespan.

## Supplementary Material

gbaf189_Supplementary_Data

## Data Availability

To access the Lothian Birth Cohort data, see https://lothian-birth-cohorts.ed.ac.uk/data-access-collaboration. The R code for all structural equation models is included in the Supplementary Material. The study was not preregistered.
