# Supplementary material for: Cognitive dedifferentiation in later life: longitudinal findings from the Lothian Birth Cohort 1936
Source: J Gerontol B Psychol Sci Soc Sci. 2025 Oct 4;81(1):gbaf189. doi: 10.1093/geronb/gbaf189 (PMC12779353; doi:10.1093/geronb/gbaf189)
Supplement: gbaf189_Supplementary_Data [file gbaf189_supplementary_data.zip › JGPS suppl Moodie, Corley, Deary, & Cox.docx]

***The Journals of Gerontology, Series B: Psychological Sciences and Social Sciences* Supplementary Material: Moodie, Corley, Deary, & Cox. Cognitive dedifferentiation in later life: Longitudinal findings from the Lothian Birth Cohort 1936.**

# **Supplementary Figures**


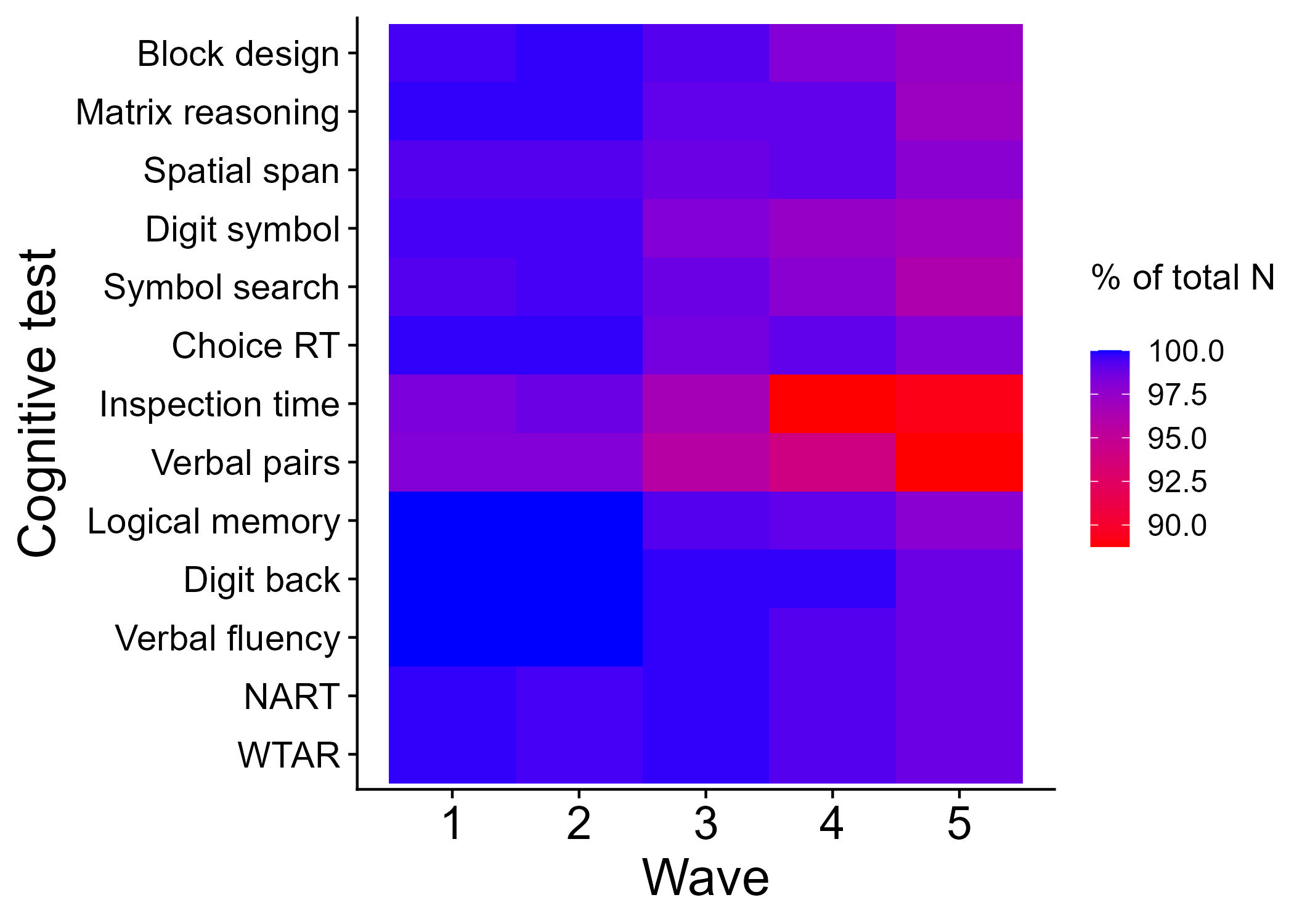


*Supplementary Figure 1. Completeness pattern of the cognitive test data across waves (total sample N = 418). The lowest scores are for ittotal_squared_w4 and vpatotal_w4, which have 89% (N = 371) of the total sample.*


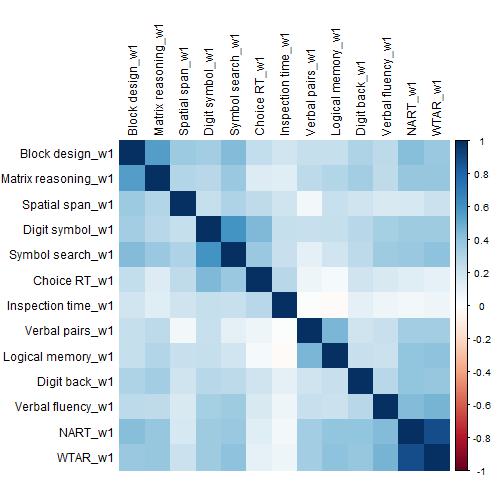

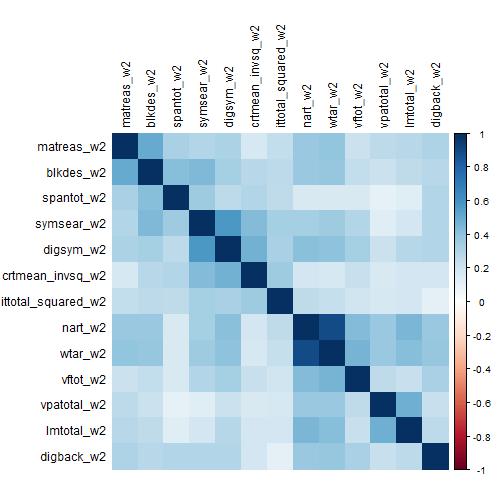


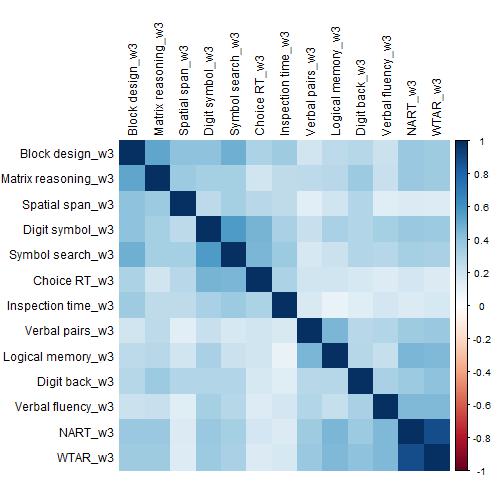

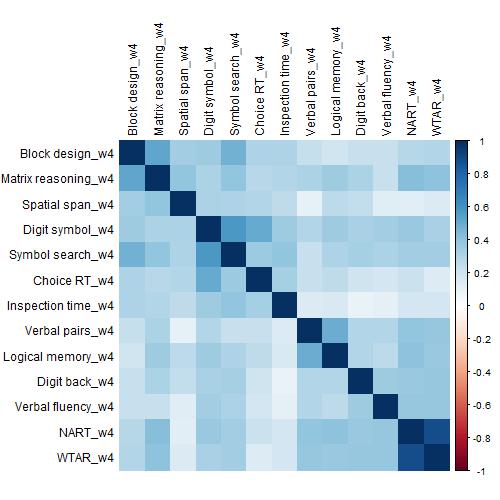


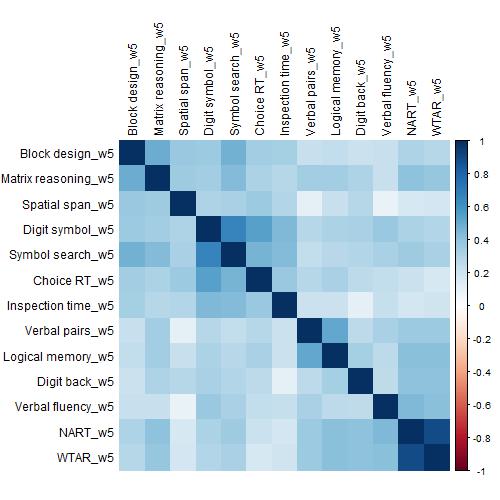


*Supplementary Figure 2. Correlation plots for the 13 cognitive test scores over five waves.*


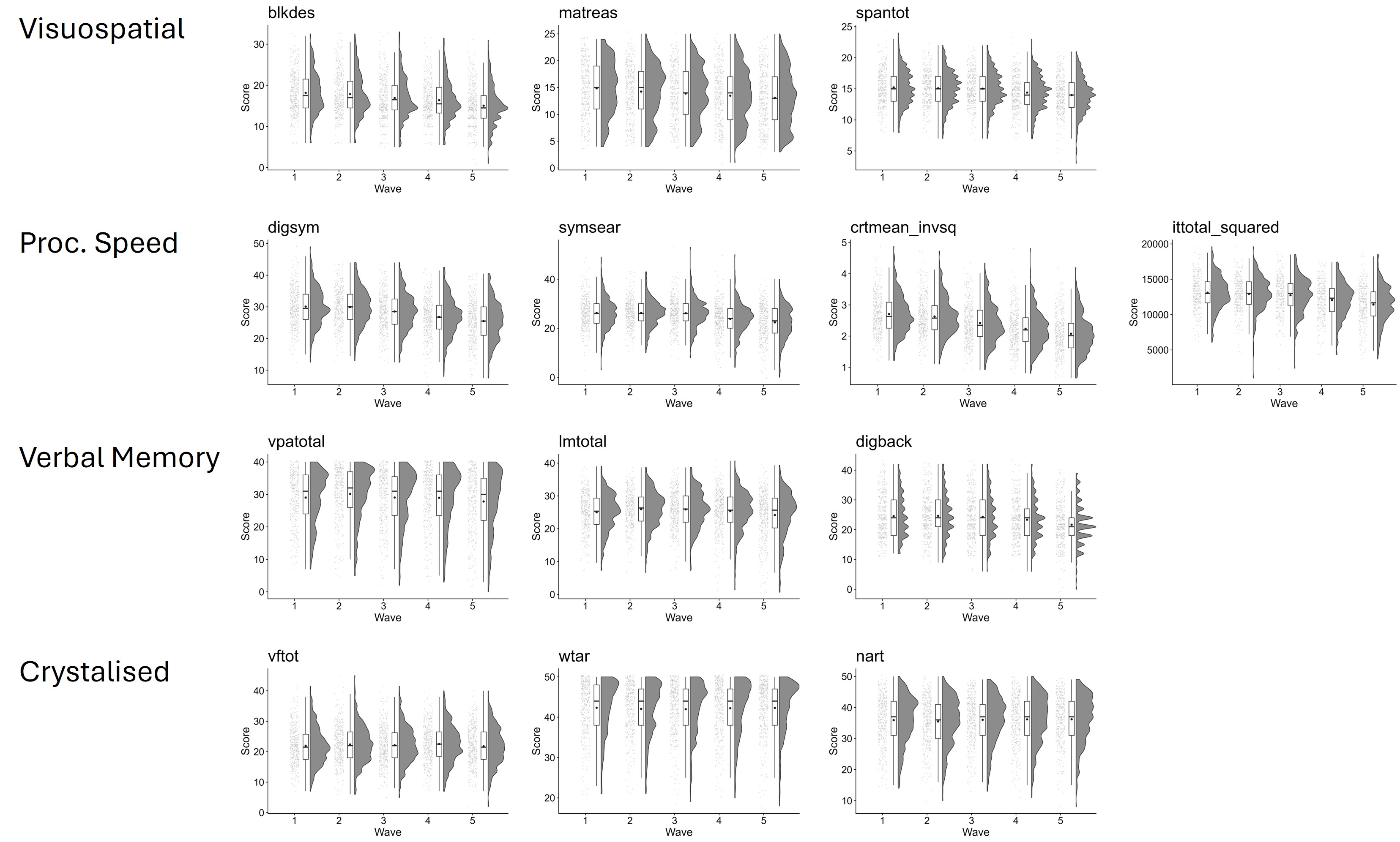


*Supplementary Figure 3. Raincloud plots for each of the cognitive test’s scores over waves 1 to 5 for people with complete data (max N = 418)*

*
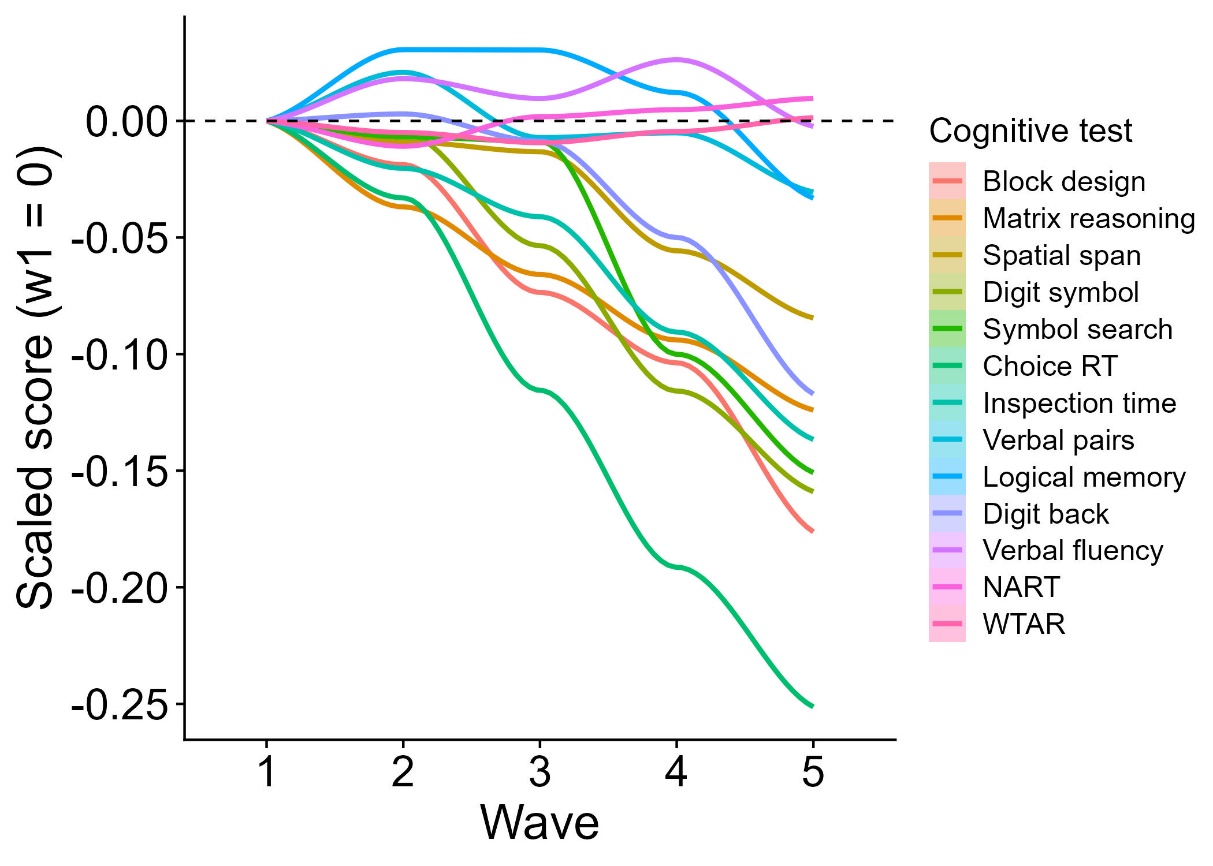
*

*Supplementary Figure 4. Means of the raw scores for each of the 13 cognitive tests by wave, all centered by setting the mean of w1 to zero. The horizontal line at 0 represents no change, values below it show negative change (declined cognitive performance), and above it signify positive change (improved cognitive performance). method = loess.*

*
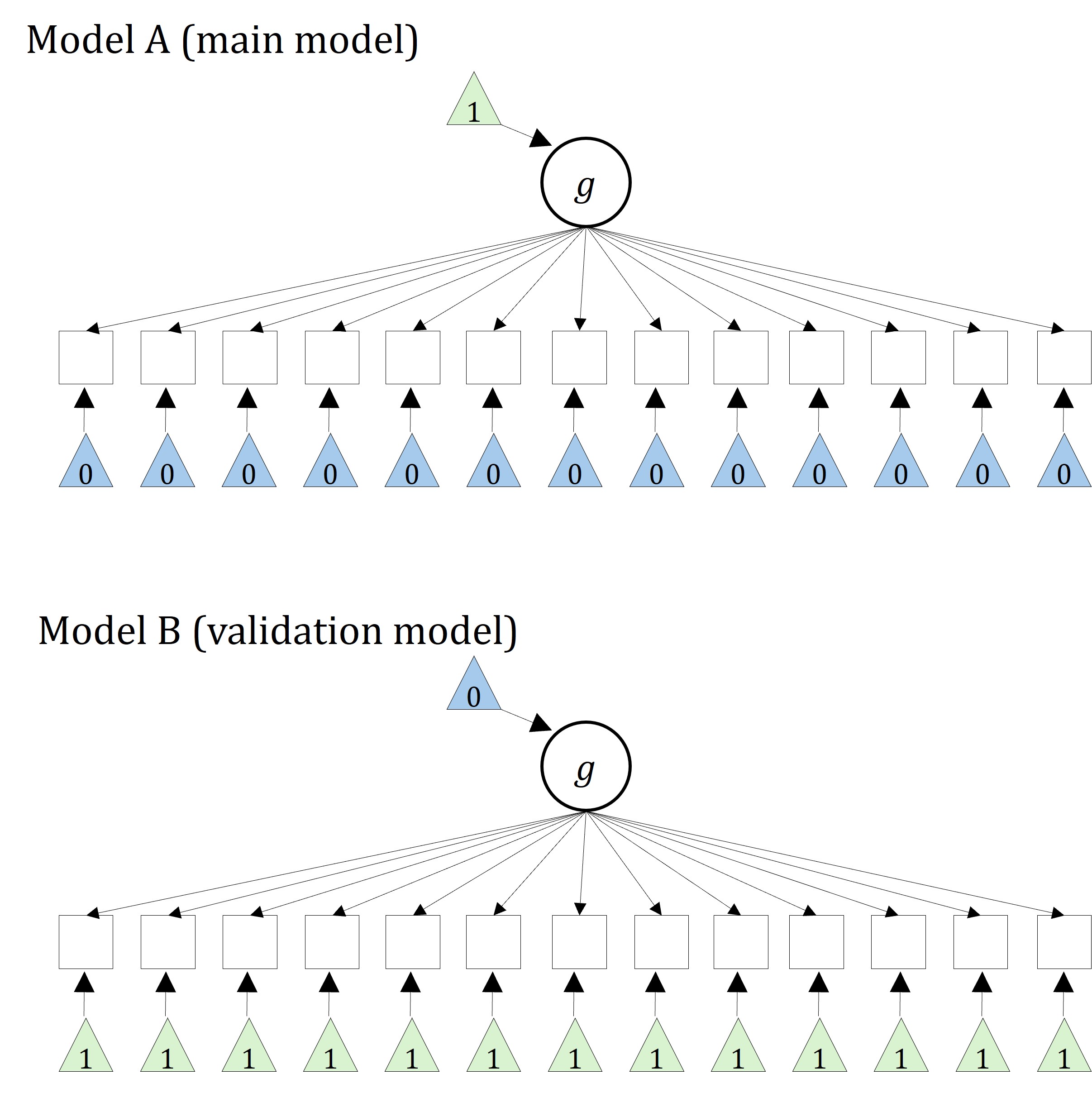
*

*Supplementary Figure 5. Illustrations of the two main model types (Model A and Model B). In Model A, the latent g intercept is estimated, and the cognitive test intercepts are set to 0; in Model B, the latent g intercept is set to 0, and the cognitive test intercepts are estimated. We ran Model A and Model B, which included all waves in the same model, and models A/B1-5, which were separate models, one per wave.*


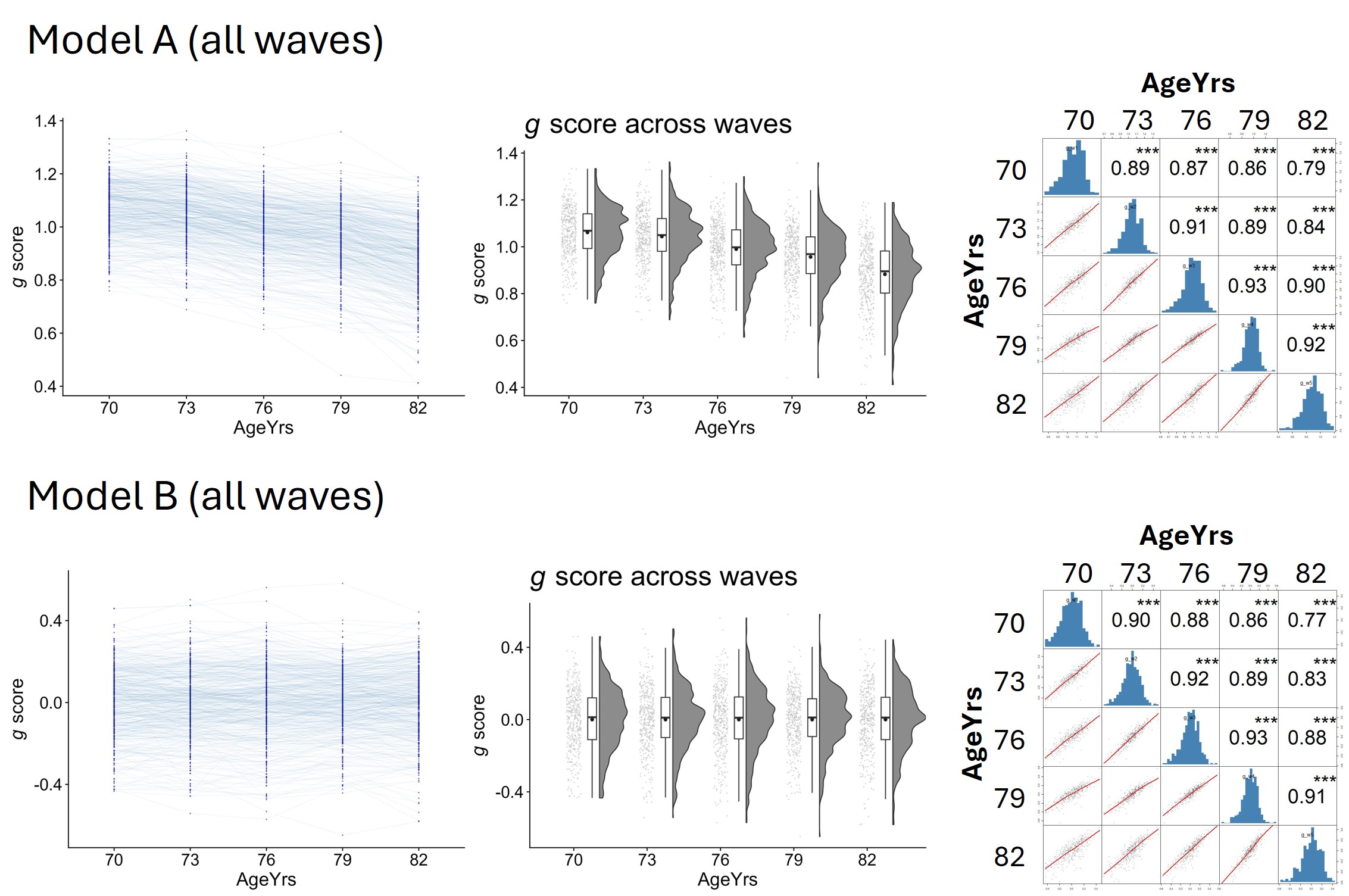


*Supplementary Figure 6. g scores across the five waves. Top: Model A (all waves) and Bottom: Model B (all waves). This figure also demonstrates why it is necessary to model g as in Model A (all waves) when the goal is to extract relative g scores across waves from a model. In Model B (all waves), all g scores are centered at zero, which means that the information about change in g across waves is limited.*

*
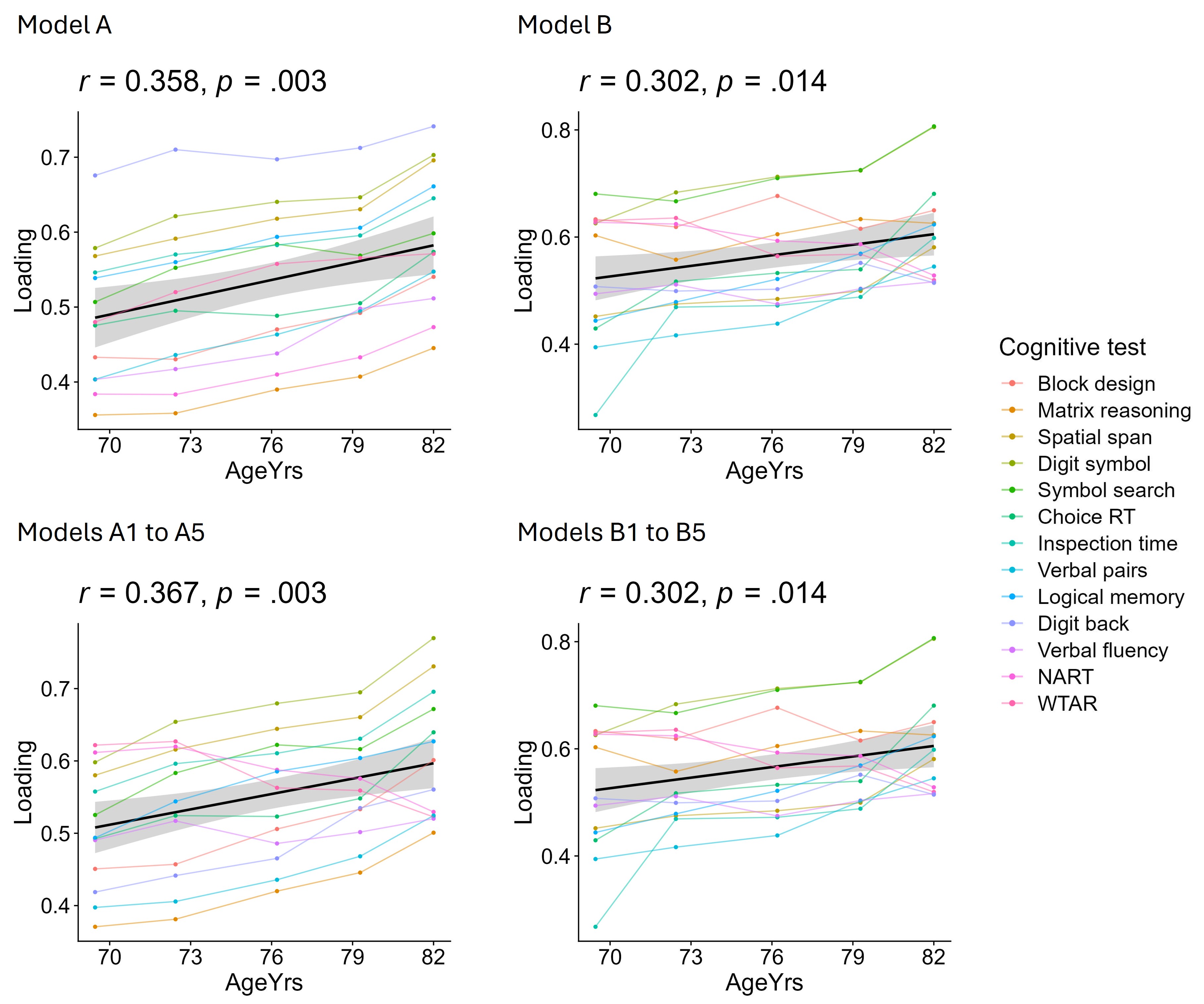
Supplementary Figure 7. Loadings on g across waves for the four different model types (A, A1 to 5, B, and B1 to 5).* The dashed horizontal line is at the intercept for each line and represents no change.


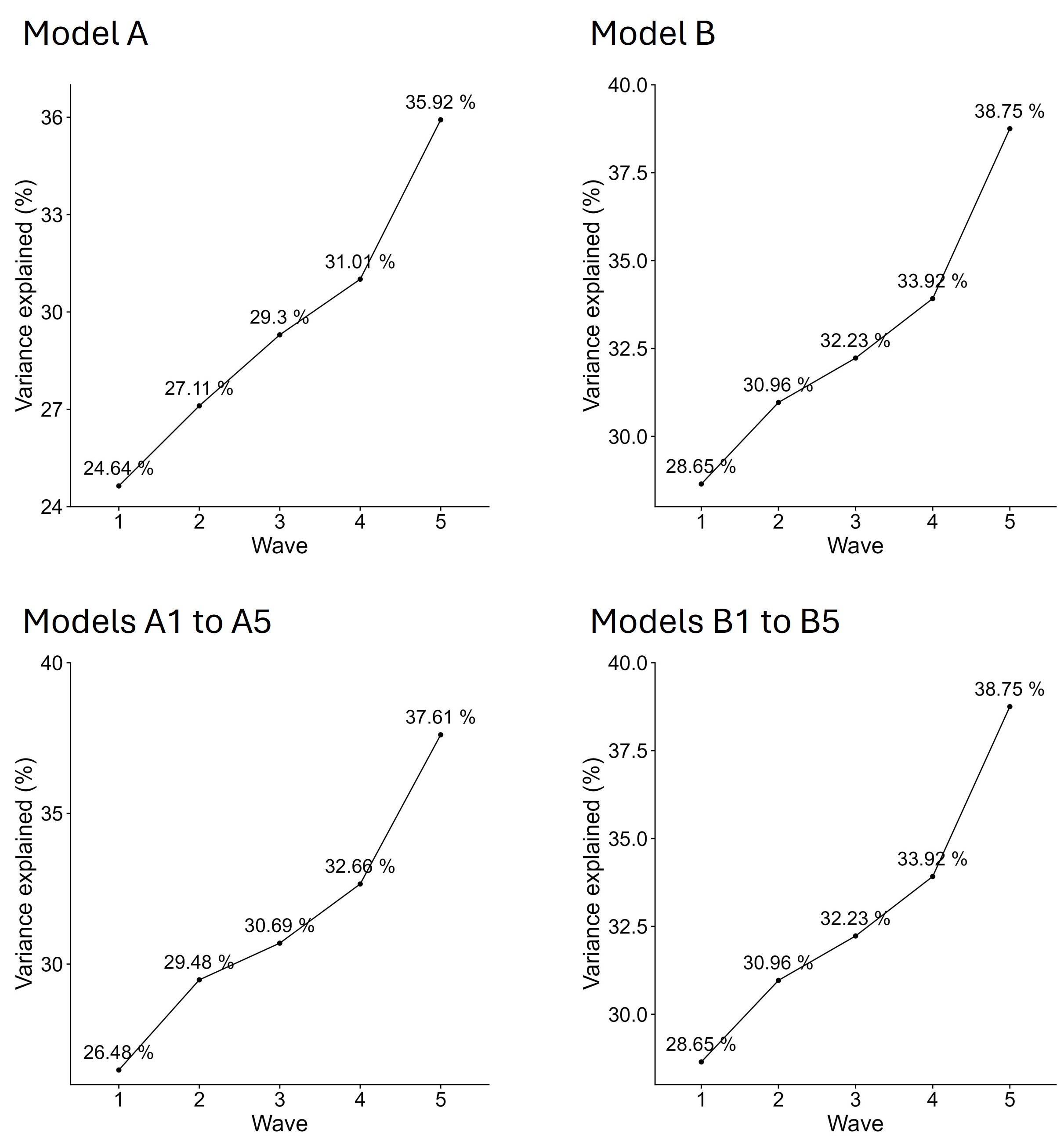


*Supplementary Figure 8. Variance explained by g across waves for the four different model types (A, A1 to 5, B, and B1 to 5).*

*
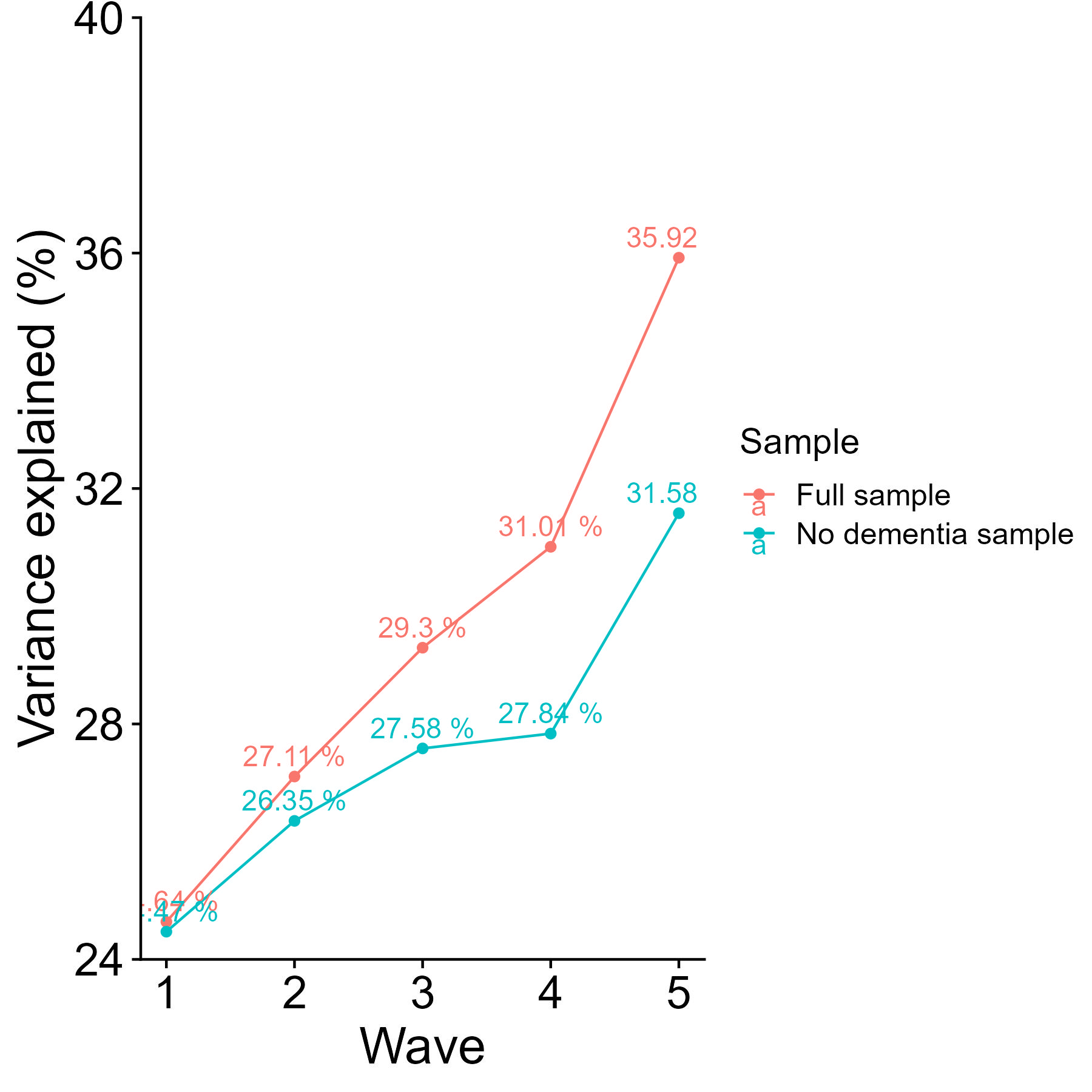

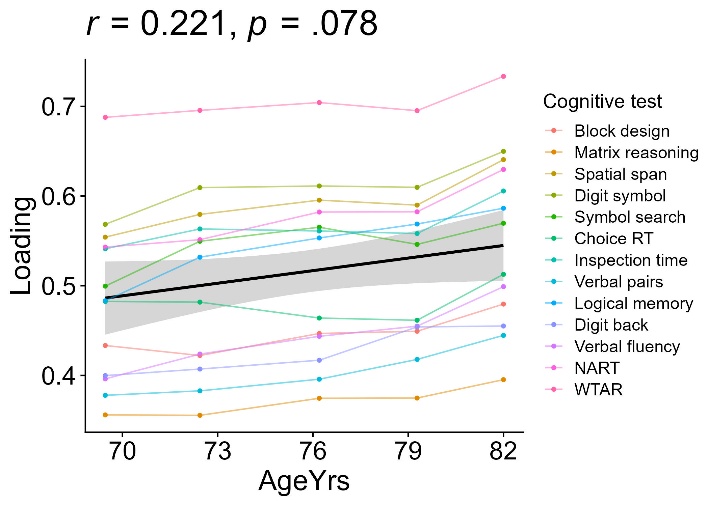
*

*Supplementary Figure 9. Sensitivity analysis: N = 360 who do not have a subsequent dementia diagnosis as of 6^th^ March 2025. Left: Variance explained by g for each of the five waves, for the full sample compared to the no dementia sample. Right: Loadings for each cognitive test across waves for the no dementia sample (to be compared with Figure 1E for the full sample).*


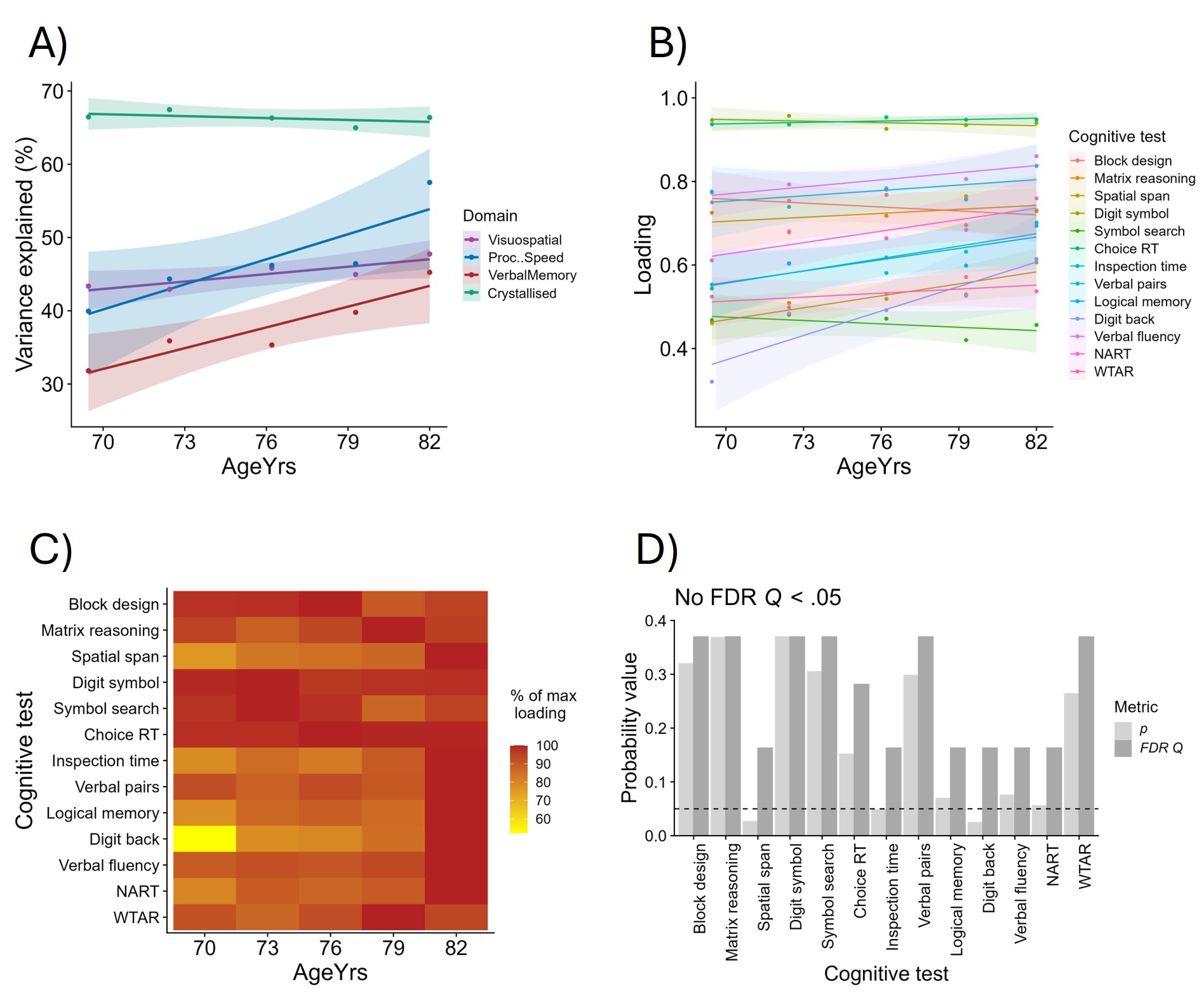


*Supplementary Figure 10. Domain-to-test results. A) shows the proportion of variance explained from domains-to-tests across waves, B) shows the domain-to-test loadings across the 5 waves, C) is a heat map showing the same data as in B for ease of pattern comparison, and D) is a bar chart showing that there were no FDR Q values < .05 for individual test-to-domain loadings by waves.*


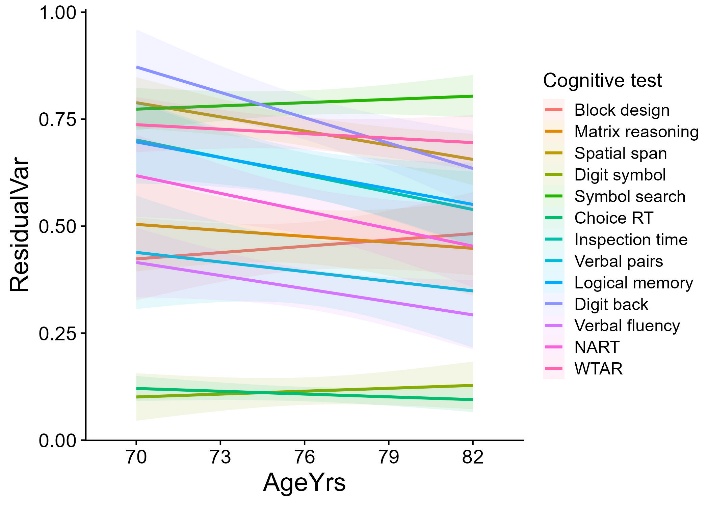

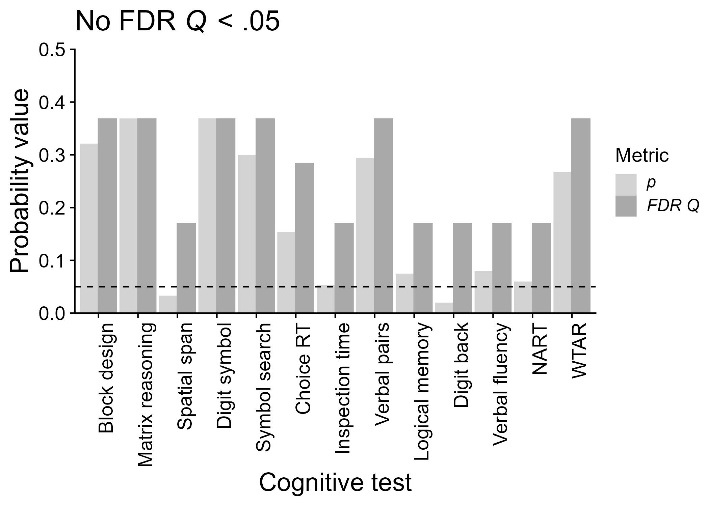


*Supplementary Figure 11. Residual variances of tests in the hierarchical analysis. Left: Residual variances for each of the 13 cognitive tests over the 5 waves; Right: Probability values of changes in residual variance across waves for each of the 13 tests.*

# **Supplementary Tables**

*Supplementary Table 1.* Associations between missingness (0 = none missing, 1 = at least one w2 to w5 missing) and cognitive test score at w1.

| Cognitive test | Estimate | SE | β | z | *p* |
| --- | --- | --- | --- | --- | --- |
| Block design | -4.023 | 0.637 | -0.188 | -6.313 | 3.97E-10 |
| Matrix reasoning | -2.153 | 0.316 | -0.203 | -6.816 | 1.55E-11 |
| Spatial span | -0.821 | 0.176 | -0.14 | -4.654 | 3.65E-06 |
| Symbol search | -2.341 | 0.39 | -0.18 | -6.003 | 2.64E-09 |
| Digit-symbol substitution | -5.618 | 0.797 | -0.209 | -7.05 | 3.17E-12 |
| Inspection time, squared | -2.836 | 0.717 | -0.122 | -3.955 | 8.17E-05 |
| Four-choice reaction time (s) (inverse square) | 0.036 | 0.005 | 0.201 | 6.746 | 2.47E-11 |
| Verbal paired associates | -3.919 | 0.584 | -0.203 | -6.714 | 3.09E-11 |
| Logical memory | -5.786 | 1.114 | -0.156 | -5.193 | 2.48E-07 |
| Digit span backwards | -0.726 | 0.14 | -0.156 | -5.197 | 2.42E-07 |
| Verbal fluency | -2.364 | 0.781 | -0.091 | -3.026 | 2.53E-03 |
| NART | -2.329 | 0.505 | -0.138 | -4.61 | 4.50E-06 |
| WTAR | -2.115 | 0.444 | -0.143 | -4.763 | 2.16E-06 |

*Supplementary Table 2. Brief descriptions of cognitive tests*

| **Domain** | **Cognitive test** | **Brief description** | **Code** |
| --- | --- | --- | --- |
| Visuospatial | Block design | Number of puzzles correct in two minutes | blkdes |
|  | Matrix reasoning | Number of puzzles correct | matreas |
|  | Spatial span | Number of block sequences correct | spantot |
| Proc. Speed | Symbol search | Number of symbols correctly detected | symsear |
|  | Digit-symbol substitution | Number of digit-symbol pairs matched | digsym |
|  | Inspection time, squared | Number of correct responses – is the left or right line longer? | ittotal |
|  | Four-choice reaction time (s) (inverse square) | Time taken to press the indicated button (out of 4 buttons) | crtmean |
| Verbal Memory | Verbal paired associates | Number of novel word pairs matched in recall (total from immediate and delayed tests) | vpatotal |
|  | Logical memory | Number of story details recalled (out of a total possible of 25) Total from immediate and delayed tests | lmtotal |
|  | Digit span backwards | Max number of a string of numbers recalled in reverse | digback |
| Crystallised | Verbal fluency | Number of words recalled beginning with C, F and L in 3x1 minute | vftot |
|  | NART | Number of words pronounced correctly | nart |
|  | WTAR | Number of words pronounced correctly | wtar |

*Supplementary Table 3. Descriptive statistics for each of the 13 cognitive tests included in the models.*

| **Cognitive test** | **N** | **Mean** | **SD** | **Min** | **Max** | **Skew** | **Kurtosis** |
| --- | --- | --- | --- | --- | --- | --- | --- |
| blkdes_w1 | 416 | 36.06 | 9.98 | 12 | 65 | 0.21 | -0.17 |
| blkdes_w2 | 417 | 35.42 | 10.28 | 10 | 65 | 0.47 | -0.05 |
| blkdes_w3 | 415 | 33.55 | 9.89 | 10 | 66 | 0.36 | 0.33 |
| blkdes_w4 | 410 | 32.52 | 9.56 | 0 | 63 | 0.38 | 0.55 |
| blkdes_w5 | 407 | 30.05 | 9.61 | 2 | 62 | 0.29 | 0.26 |
| matreas_w1 | 417 | 14.77 | 4.99 | 4 | 24 | -0.14 | -0.88 |
| matreas_w2 | 417 | 14.25 | 4.92 | 4 | 25 | -0.23 | -0.86 |
| matreas_w3 | 414 | 13.84 | 4.85 | 4 | 25 | -0.09 | -0.88 |
| matreas_w4 | 414 | 13.44 | 4.96 | 1 | 25 | 0.01 | -0.86 |
| matreas_w5 | 406 | 13.02 | 5.2 | 3 | 25 | 0.08 | -0.94 |
| spantot_w1 | 415 | 15.2 | 2.79 | 8 | 24 | 0.1 | -0.2 |
| spantot_w2 | 415 | 15.06 | 2.65 | 7 | 22 | -0.09 | -0.17 |
| spantot_w3 | 413 | 15 | 2.7 | 7 | 22 | -0.04 | -0.22 |
| spantot_w4 | 414 | 14.37 | 2.77 | 7 | 23 | -0.1 | -0.13 |
| spantot_w5 | 409 | 13.95 | 2.85 | 3 | 21 | -0.23 | 0.4 |
| symsear_w1 | 415 | 26.1 | 6.28 | 3 | 49 | 0.11 | 0.7 |
| symsear_w2 | 416 | 25.93 | 5.79 | 4 | 43 | -0.21 | 1.03 |
| symsear_w3 | 413 | 25.89 | 6.04 | 6 | 53 | 0 | 1.08 |
| symsear_w4 | 409 | 23.61 | 6.23 | 4 | 50 | -0.19 | 1.14 |
| symsear_w5 | 402 | 22.34 | 6.81 | 0 | 40 | -0.45 | 0.25 |
| digsym_w1 | 416 | 60 | 11.98 | 25 | 98 | 0.14 | 0.07 |
| digsym_w2 | 416 | 59.7 | 11.56 | 26 | 93 | 0.01 | -0.2 |
| digsym_w3 | 410 | 56.99 | 11.74 | 15 | 88 | -0.09 | 0.13 |
| digsym_w4 | 407 | 53.5 | 11.99 | 15 | 85 | -0.14 | 0.14 |
| digsym_w5 | 405 | 51.08 | 12.64 | 15 | 81 | -0.07 | -0.27 |
| crtmean_invsq_w1 | 417 | 2.69 | 0.62 | 0.78 | 4.87 | 0.39 | 0.28 |
| crtmean_invsq_w2 | 417 | 2.61 | 0.62 | 1.11 | 4.73 | 0.45 | 0.22 |
| crtmean_invsq_w3 | 412 | 2.41 | 0.63 | 0.77 | 4.34 | 0.35 | -0.1 |
| crtmean_invsq_w4 | 414 | 2.22 | 0.61 | 0.81 | 4.81 | 0.43 | 0.37 |
| crtmean_invsq_w5 | 410 | 2.07 | 0.62 | 0.66 | 4.2 | 0.37 | -0.13 |
| ittotal_squared_w1 | 411 | 13109.7 | 2244.34 | 6084 | 19600 | -0.12 | 0.16 |
| ittotal_squared_w2 | 413 | 12854.6 | 2426.98 | 1024 | 19600 | -0.67 | 1.56 |
| ittotal_squared_w3 | 404 | 12594.2 | 2483.16 | 2401 | 18496 | -0.37 | 0.19 |
| ittotal_squared_w4 | 371 | 11973.9 | 2468.97 | 4356 | 17424 | -0.36 | 0.04 |
| ittotal_squared_w5 | 373 | 11395.6 | 2602.11 | 3721 | 18496 | -0.23 | 0.04 |
| vpatotal_w1 | 410 | 28.36 | 8.25 | 6 | 40 | -0.62 | -0.56 |
| vpatotal_w2 | 410 | 28.95 | 8.83 | 3 | 40 | -0.93 | 0.04 |
| vpatotal_w3 | 400 | 28.16 | 8.77 | 1 | 40 | -0.76 | -0.16 |
| vpatotal_w4 | 393 | 28.21 | 9.03 | 3 | 40 | -0.76 | -0.25 |
| vpa_total_w5 | 371 | 27.49 | 9.53 | 0 | 40 | -0.78 | -0.19 |
| lmtotal_w1 | 418 | 74.88 | 17.12 | 21 | 117 | -0.44 | -0.01 |
| lmtotal_w2 | 418 | 77.19 | 16.79 | 20 | 116 | -0.47 | 0.27 |
| lmtotal_w3 | 415 | 77.18 | 17.17 | 22 | 116 | -0.35 | 0.04 |
| lmtotal_w4 | 414 | 75.8 | 18.3 | 4 | 122 | -0.66 | 0.86 |
| lmtotal_w5 | 409 | 72.38 | 21.55 | 2 | 118 | -0.81 | 0.47 |
| digback_w1 | 418 | 8.16 | 2.35 | 4 | 14 | 0.41 | -0.58 |
| digback_w2 | 418 | 8.18 | 2.35 | 2 | 14 | 0.29 | -0.22 |
| digback_w3 | 417 | 8.09 | 2.41 | 2 | 14 | 0.22 | -0.48 |
| digback_w4 | 417 | 7.76 | 2.19 | 2 | 14 | 0.36 | -0.22 |
| digback_w5 | 413 | 7.22 | 2.33 | 0 | 13 | 0.12 | 0.38 |
| vftot_w1 | 418 | 43.65 | 12.49 | 14 | 83 | 0.37 | -0.09 |
| vftot_w2 | 418 | 44.47 | 12.24 | 6 | 90 | 0.15 | 0.35 |
| vftot_w3 | 417 | 44.08 | 12.18 | 10 | 83 | 0.15 | 0.07 |
| vftot_w4 | 415 | 44.83 | 12.45 | 14 | 80 | 0.06 | -0.25 |
| vftot_w5 | 413 | 43.55 | 12.68 | 4 | 80 | -0.02 | -0.23 |
| nart_w1 | 417 | 35.85 | 7.74 | 14 | 50 | -0.59 | -0.24 |
| nart_w2 | 416 | 35.46 | 7.62 | 10 | 50 | -0.55 | -0.22 |
| nart_total_w3 | 417 | 35.92 | 7.62 | 12 | 49 | -0.6 | -0.1 |
| nart_total_w4 | 415 | 36.03 | 7.94 | 6 | 50 | -0.65 | 0.11 |
| nart_total_w5 | 413 | 36.2 | 7.73 | 8 | 49 | -0.68 | 0.1 |
| wtar_w1 | 417 | 42.27 | 6.65 | 14 | 50 | -1.07 | 0.86 |
| wtar_w2 | 416 | 42.06 | 6.27 | 21 | 50 | -0.86 | 0.2 |
| wtar_total_w3 | 417 | 41.88 | 6.59 | 9 | 50 | -1.13 | 1.49 |
| wtar_total_w4 | 415 | 42.08 | 6.7 | 15 | 50 | -1.07 | 0.83 |
| wtar_total_w5 | 413 | 42.33 | 6.5 | 18 | 50 | -1.17 | 1.08 |

Note These variables were then scaled before inclusion in the models.

*Supplementary Table 4. Model fits for different g models.*

| **Model number** | **Model type** | **Waves** | **CFI** | **TLI** | **RMSEA** | **SRMR** |
| --- | --- | --- | --- | --- | --- | --- |
| A | *g* intercept estimated, cognitive tests intercepts fixed to 0 | All waves | 0.296 | 0.292 | 0.149 | 0.340 |
| A1 |  | w1 | 0.764 | 0.748 | 0.120 | 0.141 |
| A2 |  | w2 | 0.818 | 0.806 | 0.110 | 0.115 |
| A3 |  | w3 | 0.816 | 0.803 | 0.111 | 0.126 |
| A4 |  | w4 | 0.840 | 0.829 | 0.106 | 0.124 |
| A5 |  | w5 | 0.842 | 0.831 | 0.112 | 0.131 |
| B | *g* intercept fixed to 0, cognitive tests intercepts estimated | All waves | 0.337 | 0.314 | 0.147 | 0.334 |
| B1 |  | w1 | 0.843 | 0.809 | 0.105 | 0.075 |
| B2 |  | w2 | 0.858 | 0.827 | 0.104 | 0.074 |
| B3 |  | w3 | 0.870 | 0.842 | 0.100 | 0.071 |
| B4 |  | w4 | 0.887 | 0.863 | 0.095 | 0.064 |
| B5 |  | w5 | 0.891 | 0.867 | 0.100 | 0.068 |
| Hierarchical Model | *g* intercept fixed to 0, cognitive tests intercepts estimated | All waves | 0.360 | 0.333 | 0.146 | 0.334 |

*Supplementary Table 5. Standardized solution of g model, model loadings: Model A (all waves)*

| **lhs** | **op** | **rhs** | **est.std** | **se** | **z** | **pvalue** | **ci.lower** | **ci.upper** |
| --- | --- | --- | --- | --- | --- | --- | --- | --- |
| g_w1 | =~ | blkdes_w1 | 0.433 | 0.021 | 20.514 | 0.000 | 0.392 | 0.474 |
| g_w1 | =~ | matreas_w1 | 0.356 | 0.019 | 19.100 | 0.000 | 0.319 | 0.392 |
| g_w1 | =~ | spantot_w1 | 0.568 | 0.022 | 25.459 | 0.000 | 0.524 | 0.612 |
| g_w1 | =~ | digsym_w1 | 0.579 | 0.023 | 24.678 | 0.000 | 0.533 | 0.625 |
| g_w1 | =~ | symsear_w1 | 0.507 | 0.023 | 22.005 | 0.000 | 0.462 | 0.552 |
| g_w1 | =~ | crtmean_invsq_w1 | 0.476 | 0.021 | 22.118 | 0.000 | 0.433 | 0.518 |
| g_w1 | =~ | ittotal_squared_w1 | 0.546 | 0.021 | 25.411 | 0.000 | 0.504 | 0.588 |
| g_w1 | =~ | vpatotal_w1 | 0.384 | 0.019 | 19.967 | 0.000 | 0.346 | 0.422 |
| g_w1 | =~ | lmtotal_w1 | 0.480 | 0.022 | 22.273 | 0.000 | 0.438 | 0.522 |
| g_w1 | =~ | digback_w1 | 0.403 | 0.020 | 20.189 | 0.000 | 0.364 | 0.443 |
| g_w1 | =~ | vftot_w1 | 0.403 | 0.020 | 20.218 | 0.000 | 0.364 | 0.443 |
| g_w1 | =~ | nart_w1 | 0.539 | 0.023 | 23.278 | 0.000 | 0.493 | 0.584 |
| g_w1 | =~ | wtar_w1 | 0.676 | 0.022 | 30.183 | 0.000 | 0.632 | 0.720 |
| g_w2 | =~ | blkdes_w2 | 0.430 | 0.021 | 20.916 | 0.000 | 0.390 | 0.471 |
| g_w2 | =~ | matreas_w2 | 0.358 | 0.018 | 19.593 | 0.000 | 0.322 | 0.394 |
| g_w2 | =~ | spantot_w2 | 0.591 | 0.022 | 27.084 | 0.000 | 0.549 | 0.634 |
| g_w2 | =~ | digsym_w2 | 0.621 | 0.023 | 26.983 | 0.000 | 0.576 | 0.666 |
| g_w2 | =~ | symsear_w2 | 0.552 | 0.023 | 24.143 | 0.000 | 0.508 | 0.597 |
| g_w2 | =~ | crtmean_invsq_w2 | 0.495 | 0.022 | 22.847 | 0.000 | 0.453 | 0.538 |
| g_w2 | =~ | ittotal_squared_w2 | 0.570 | 0.022 | 25.969 | 0.000 | 0.527 | 0.613 |
| g_w2 | =~ | vpatotal_w2 | 0.383 | 0.019 | 20.276 | 0.000 | 0.346 | 0.420 |
| g_w2 | =~ | lmtotal_w2 | 0.520 | 0.022 | 23.931 | 0.000 | 0.477 | 0.563 |
| g_w2 | =~ | digback_w2 | 0.417 | 0.020 | 20.942 | 0.000 | 0.378 | 0.456 |
| g_w2 | =~ | vftot_w2 | 0.436 | 0.020 | 21.335 | 0.000 | 0.396 | 0.476 |
| g_w2 | =~ | nart_w2 | 0.560 | 0.023 | 24.661 | 0.000 | 0.515 | 0.604 |
| g_w2 | =~ | wtar_w2 | 0.710 | 0.021 | 34.046 | 0.000 | 0.669 | 0.751 |
| g_w3 | =~ | blkdes_w3 | 0.470 | 0.022 | 21.765 | 0.000 | 0.428 | 0.513 |
| g_w3 | =~ | matreas_w3 | 0.390 | 0.019 | 20.101 | 0.000 | 0.352 | 0.428 |
| g_w3 | =~ | spantot_w3 | 0.618 | 0.022 | 28.722 | 0.000 | 0.576 | 0.660 |
| g_w3 | =~ | digsym_w3 | 0.640 | 0.023 | 27.905 | 0.000 | 0.595 | 0.685 |
| g_w3 | =~ | symsear_w3 | 0.584 | 0.023 | 25.268 | 0.000 | 0.539 | 0.629 |
| g_w3 | =~ | crtmean_invsq_w3 | 0.488 | 0.022 | 22.645 | 0.000 | 0.446 | 0.531 |
| g_w3 | =~ | ittotal_squared_w3 | 0.583 | 0.022 | 26.597 | 0.000 | 0.540 | 0.626 |
| g_w3 | =~ | vpatotal_w3 | 0.410 | 0.020 | 20.739 | 0.000 | 0.371 | 0.449 |
| g_w3 | =~ | lmtotal_w3 | 0.558 | 0.022 | 25.265 | 0.000 | 0.514 | 0.601 |
| g_w3 | =~ | digback_w3 | 0.438 | 0.020 | 21.468 | 0.000 | 0.398 | 0.478 |
| g_w3 | =~ | vftot_w3 | 0.463 | 0.021 | 22.183 | 0.000 | 0.422 | 0.504 |
| g_w3 | =~ | nart_total_w3 | 0.594 | 0.022 | 26.555 | 0.000 | 0.550 | 0.638 |
| g_w3 | =~ | wtar_total_w3 | 0.697 | 0.020 | 34.046 | 0.000 | 0.657 | 0.737 |
| g_w4 | =~ | blkdes_w4 | 0.492 | 0.022 | 22.607 | 0.000 | 0.450 | 0.535 |
| g_w4 | =~ | matreas_w4 | 0.407 | 0.020 | 20.396 | 0.000 | 0.368 | 0.446 |
| g_w4 | =~ | spantot_w4 | 0.630 | 0.021 | 29.466 | 0.000 | 0.589 | 0.672 |
| g_w4 | =~ | digsym_w4 | 0.646 | 0.023 | 28.249 | 0.000 | 0.602 | 0.691 |
| g_w4 | =~ | symsear_w4 | 0.569 | 0.023 | 24.664 | 0.000 | 0.523 | 0.614 |
| g_w4 | =~ | crtmean_invsq_w4 | 0.505 | 0.022 | 23.218 | 0.000 | 0.463 | 0.548 |
| g_w4 | =~ | ittotal_squared_w4 | 0.596 | 0.022 | 26.656 | 0.000 | 0.552 | 0.639 |
| g_w4 | =~ | vpatotal_w4 | 0.433 | 0.021 | 21.099 | 0.000 | 0.393 | 0.473 |
| g_w4 | =~ | lmtotal_w4 | 0.566 | 0.022 | 25.473 | 0.000 | 0.522 | 0.609 |
| g_w4 | =~ | digback_w4 | 0.498 | 0.022 | 23.075 | 0.000 | 0.456 | 0.540 |
| g_w4 | =~ | vftot_w4 | 0.494 | 0.021 | 23.088 | 0.000 | 0.452 | 0.536 |
| g_w4 | =~ | nart_total_w4 | 0.606 | 0.022 | 27.446 | 0.000 | 0.563 | 0.649 |
| g_w4 | =~ | wtar_total_w4 | 0.712 | 0.020 | 36.055 | 0.000 | 0.674 | 0.751 |
| g_w5 | =~ | blkdes_w5 | 0.540 | 0.022 | 24.105 | 0.000 | 0.496 | 0.584 |
| g_w5 | =~ | matreas_w5 | 0.445 | 0.021 | 21.195 | 0.000 | 0.404 | 0.486 |
| g_w5 | =~ | spantot_w5 | 0.696 | 0.020 | 34.145 | 0.000 | 0.656 | 0.736 |
| g_w5 | =~ | digsym_w5 | 0.703 | 0.022 | 31.729 | 0.000 | 0.660 | 0.746 |
| g_w5 | =~ | symsear_w5 | 0.598 | 0.023 | 25.552 | 0.000 | 0.553 | 0.644 |
| g_w5 | =~ | crtmean_invsq_w5 | 0.574 | 0.023 | 25.234 | 0.000 | 0.529 | 0.618 |
| g_w5 | =~ | ittotal_squared_w5 | 0.645 | 0.022 | 29.142 | 0.000 | 0.602 | 0.689 |
| g_w5 | =~ | vpa_total_w5 | 0.473 | 0.022 | 21.797 | 0.000 | 0.431 | 0.516 |
| g_w5 | =~ | lmtotal_w5 | 0.571 | 0.023 | 25.367 | 0.000 | 0.527 | 0.615 |
| g_w5 | =~ | digback_w5 | 0.512 | 0.022 | 23.604 | 0.000 | 0.469 | 0.554 |
| g_w5 | =~ | vftot_w5 | 0.547 | 0.022 | 24.994 | 0.000 | 0.504 | 0.590 |
| g_w5 | =~ | nart_total_w5 | 0.661 | 0.021 | 31.870 | 0.000 | 0.620 | 0.702 |
| g_w5 | =~ | wtar_total_w5 | 0.741 | 0.018 | 40.923 | 0.000 | 0.706 | 0.777 |
| nart_w1 | ~~ | wtar_w1 | 0.807 | 0.019 | 42.627 | 0.000 | 0.770 | 0.844 |
| nart_w2 | ~~ | wtar_w2 | 0.802 | 0.020 | 41.125 | 0.000 | 0.764 | 0.841 |
| nart_total_w3 | ~~ | wtar_total_w3 | 0.795 | 0.020 | 40.194 | 0.000 | 0.756 | 0.834 |
| nart_total_w4 | ~~ | wtar_total_w4 | 0.799 | 0.020 | 40.955 | 0.000 | 0.761 | 0.837 |
| nart_total_w5 | ~~ | wtar_total_w5 | 0.824 | 0.017 | 47.408 | 0.000 | 0.790 | 0.859 |

*Supplementary Table 6. Standardized solution of g model, model loadings: Model A1, A2, A3, A4, and A5 (separate models for each wave)*

| **lhs** | **op** | **rhs** | **est.std** | **se** | **z** | **pvalue** | **ci.lower** | **ci.upper** |
| --- | --- | --- | --- | --- | --- | --- | --- | --- |
| g_w1 | =~ | blkdes_w1 | 0.451 | 0.022 | 20.118 | 0.000 | 0.407 | 0.495 |
| g_w1 | =~ | matreas_w1 | 0.371 | 0.020 | 18.674 | 0.000 | 0.332 | 0.410 |
| g_w1 | =~ | spantot_w1 | 0.580 | 0.023 | 25.144 | 0.000 | 0.535 | 0.625 |
| g_w1 | =~ | digsym_w1 | 0.598 | 0.024 | 24.428 | 0.000 | 0.550 | 0.646 |
| g_w1 | =~ | symsear_w1 | 0.525 | 0.024 | 21.550 | 0.000 | 0.478 | 0.573 |
| g_w1 | =~ | crtmean_invsq_w1 | 0.492 | 0.023 | 21.628 | 0.000 | 0.448 | 0.537 |
| g_w1 | =~ | ittotal_squared_w1 | 0.558 | 0.022 | 25.193 | 0.000 | 0.514 | 0.601 |
| g_w1 | =~ | vpatotal_w1 | 0.397 | 0.020 | 19.726 | 0.000 | 0.358 | 0.437 |
| g_w1 | =~ | lmtotal_w1 | 0.494 | 0.022 | 22.188 | 0.000 | 0.450 | 0.537 |
| g_w1 | =~ | digback_w1 | 0.419 | 0.021 | 19.905 | 0.000 | 0.377 | 0.460 |
| g_w1 | =~ | vftot_w1 | 0.491 | 0.043 | 11.308 | 0.000 | 0.406 | 0.576 |
| g_w1 | =~ | nart_w1 | 0.612 | 0.038 | 16.174 | 0.000 | 0.538 | 0.686 |
| g_w1 | =~ | wtar_w1 | 0.622 | 0.037 | 16.685 | 0.000 | 0.549 | 0.695 |
| nart_w1 | ~~ | wtar_w1 | 0.820 | 0.018 | 46.070 | 0.000 | 0.785 | 0.855 |
| g_w2 | =~ | blkdes_w2 | 0.457 | 0.022 | 21.115 | 0.000 | 0.415 | 0.500 |
| g_w2 | =~ | matreas_w2 | 0.381 | 0.019 | 19.649 | 0.000 | 0.343 | 0.419 |
| g_w2 | =~ | spantot_w2 | 0.616 | 0.022 | 27.938 | 0.000 | 0.573 | 0.659 |
| g_w2 | =~ | digsym_w2 | 0.654 | 0.023 | 28.222 | 0.000 | 0.609 | 0.700 |
| g_w2 | =~ | symsear_w2 | 0.583 | 0.024 | 24.716 | 0.000 | 0.537 | 0.630 |
| g_w2 | =~ | crtmean_invsq_w2 | 0.524 | 0.023 | 23.223 | 0.000 | 0.480 | 0.569 |
| g_w2 | =~ | ittotal_squared_w2 | 0.596 | 0.022 | 26.849 | 0.000 | 0.553 | 0.640 |
| g_w2 | =~ | vpatotal_w2 | 0.406 | 0.020 | 20.498 | 0.000 | 0.367 | 0.444 |
| g_w2 | =~ | lmtotal_w2 | 0.544 | 0.022 | 24.669 | 0.000 | 0.501 | 0.587 |
| g_w2 | =~ | digback_w2 | 0.441 | 0.021 | 21.228 | 0.000 | 0.401 | 0.482 |
| g_w2 | =~ | vftot_w2 | 0.517 | 0.041 | 12.598 | 0.000 | 0.437 | 0.598 |
| g_w2 | =~ | nart_w2 | 0.620 | 0.036 | 17.166 | 0.000 | 0.549 | 0.690 |
| g_w2 | =~ | wtar_w2 | 0.627 | 0.036 | 17.545 | 0.000 | 0.557 | 0.697 |
| nart_w2 | ~~ | wtar_w2 | 0.832 | 0.016 | 50.448 | 0.000 | 0.799 | 0.864 |
| g_w3 | =~ | blkdes_w3 | 0.506 | 0.023 | 22.458 | 0.000 | 0.462 | 0.550 |
| g_w3 | =~ | matreas_w3 | 0.420 | 0.020 | 20.495 | 0.000 | 0.380 | 0.460 |
| g_w3 | =~ | spantot_w3 | 0.644 | 0.021 | 30.273 | 0.000 | 0.603 | 0.686 |
| g_w3 | =~ | digsym_w3 | 0.680 | 0.023 | 30.059 | 0.000 | 0.635 | 0.724 |
| g_w3 | =~ | symsear_w3 | 0.622 | 0.023 | 26.598 | 0.000 | 0.576 | 0.668 |
| g_w3 | =~ | crtmean_invsq_w3 | 0.523 | 0.022 | 23.394 | 0.000 | 0.479 | 0.567 |
| g_w3 | =~ | ittotal_squared_w3 | 0.611 | 0.022 | 27.847 | 0.000 | 0.568 | 0.654 |
| g_w3 | =~ | vpatotal_w3 | 0.436 | 0.021 | 21.217 | 0.000 | 0.395 | 0.476 |
| g_w3 | =~ | lmtotal_w3 | 0.585 | 0.022 | 26.498 | 0.000 | 0.542 | 0.629 |
| g_w3 | =~ | digback_w3 | 0.465 | 0.021 | 22.052 | 0.000 | 0.424 | 0.507 |
| g_w3 | =~ | vftot_w3 | 0.486 | 0.042 | 11.512 | 0.000 | 0.403 | 0.569 |
| g_w3 | =~ | nart_total_w3 | 0.588 | 0.037 | 15.756 | 0.000 | 0.515 | 0.661 |
| g_w3 | =~ | wtar_total_w3 | 0.563 | 0.039 | 14.558 | 0.000 | 0.487 | 0.639 |
| nart_total_w3 | ~~ | wtar_total_w3 | 0.827 | 0.016 | 50.422 | 0.000 | 0.795 | 0.859 |
| g_w4 | =~ | blkdes_w4 | 0.533 | 0.022 | 23.897 | 0.000 | 0.490 | 0.577 |
| g_w4 | =~ | matreas_w4 | 0.446 | 0.021 | 21.208 | 0.000 | 0.404 | 0.487 |
| g_w4 | =~ | spantot_w4 | 0.660 | 0.021 | 31.686 | 0.000 | 0.620 | 0.701 |
| g_w4 | =~ | digsym_w4 | 0.695 | 0.022 | 31.541 | 0.000 | 0.652 | 0.738 |
| g_w4 | =~ | symsear_w4 | 0.616 | 0.023 | 26.701 | 0.000 | 0.571 | 0.661 |
| g_w4 | =~ | crtmean_invsq_w4 | 0.548 | 0.022 | 24.667 | 0.000 | 0.505 | 0.592 |
| g_w4 | =~ | ittotal_squared_w4 | 0.631 | 0.022 | 28.585 | 0.000 | 0.587 | 0.674 |
| g_w4 | =~ | vpatotal_w4 | 0.468 | 0.021 | 22.009 | 0.000 | 0.426 | 0.510 |
| g_w4 | =~ | lmtotal_w4 | 0.604 | 0.022 | 27.383 | 0.000 | 0.561 | 0.647 |
| g_w4 | =~ | digback_w4 | 0.535 | 0.022 | 24.420 | 0.000 | 0.492 | 0.578 |
| g_w4 | =~ | vftot_w4 | 0.502 | 0.041 | 12.241 | 0.000 | 0.421 | 0.582 |
| g_w4 | =~ | nart_total_w4 | 0.576 | 0.037 | 15.381 | 0.000 | 0.502 | 0.649 |
| g_w4 | =~ | wtar_total_w4 | 0.559 | 0.038 | 14.599 | 0.000 | 0.484 | 0.634 |
| nart_total_w4 | ~~ | wtar_total_w4 | 0.835 | 0.016 | 53.343 | 0.000 | 0.804 | 0.865 |
| g_w5 | =~ | blkdes_w5 | 0.601 | 0.022 | 27.209 | 0.000 | 0.558 | 0.644 |
| g_w5 | =~ | matreas_w5 | 0.501 | 0.022 | 23.054 | 0.000 | 0.458 | 0.543 |
| g_w5 | =~ | spantot_w5 | 0.731 | 0.019 | 38.845 | 0.000 | 0.694 | 0.768 |
| g_w5 | =~ | digsym_w5 | 0.770 | 0.019 | 39.645 | 0.000 | 0.732 | 0.808 |
| g_w5 | =~ | symsear_w5 | 0.672 | 0.022 | 30.180 | 0.000 | 0.628 | 0.715 |
| g_w5 | =~ | crtmean_invsq_w5 | 0.640 | 0.022 | 29.166 | 0.000 | 0.597 | 0.683 |
| g_w5 | =~ | ittotal_squared_w5 | 0.696 | 0.021 | 33.537 | 0.000 | 0.655 | 0.736 |
| g_w5 | =~ | vpa_total_w5 | 0.524 | 0.022 | 23.692 | 0.000 | 0.481 | 0.568 |
| g_w5 | =~ | lmtotal_w5 | 0.627 | 0.022 | 28.766 | 0.000 | 0.584 | 0.670 |
| g_w5 | =~ | digback_w5 | 0.560 | 0.022 | 25.859 | 0.000 | 0.518 | 0.603 |
| g_w5 | =~ | vftot_w5 | 0.520 | 0.040 | 13.016 | 0.000 | 0.442 | 0.598 |
| g_w5 | =~ | nart_total_w5 | 0.529 | 0.040 | 13.389 | 0.000 | 0.452 | 0.607 |
| g_w5 | =~ | wtar_total_w5 | 0.523 | 0.040 | 13.104 | 0.000 | 0.444 | 0.601 |
| nart_total_w5 | ~~ | wtar_total_w5 | 0.851 | 0.014 | 60.976 | 0.000 | 0.824 | 0.879 |

*Supplementary Table 7. Standardized solution of g model, model loadings: Model B (all waves)*

| **lhs** | **op** | **rhs** | **est.std** | **se** | **z** | **pvalue** | **ci.lower** | **ci.upper** |
| --- | --- | --- | --- | --- | --- | --- | --- | --- |
| g_w1 | =~ | blkdes_w1 | 0.633 | 0.036 | 17.783 | 0.000 | 0.563 | 0.703 |
| g_w1 | =~ | matreas_w1 | 0.603 | 0.037 | 16.127 | 0.000 | 0.530 | 0.676 |
| g_w1 | =~ | spantot_w1 | 0.452 | 0.044 | 10.220 | 0.000 | 0.365 | 0.538 |
| g_w1 | =~ | digsym_w1 | 0.626 | 0.037 | 16.966 | 0.000 | 0.553 | 0.698 |
| g_w1 | =~ | symsear_w1 | 0.681 | 0.034 | 20.184 | 0.000 | 0.614 | 0.747 |
| g_w1 | =~ | crtmean_invsq_w1 | 0.429 | 0.046 | 9.321 | 0.000 | 0.339 | 0.520 |
| g_w1 | =~ | ittotal_squared_w1 | 0.268 | 0.051 | 5.277 | 0.000 | 0.168 | 0.367 |
| g_w1 | =~ | vpatotal_w1 | 0.394 | 0.047 | 8.301 | 0.000 | 0.301 | 0.487 |
| g_w1 | =~ | lmtotal_w1 | 0.444 | 0.045 | 9.837 | 0.000 | 0.355 | 0.532 |
| g_w1 | =~ | digback_w1 | 0.508 | 0.042 | 12.219 | 0.000 | 0.426 | 0.589 |
| g_w1 | =~ | vftot_w1 | 0.494 | 0.042 | 11.693 | 0.000 | 0.411 | 0.577 |
| g_w1 | =~ | nart_w1 | 0.627 | 0.036 | 17.309 | 0.000 | 0.556 | 0.698 |
| g_w1 | =~ | wtar_w1 | 0.630 | 0.036 | 17.494 | 0.000 | 0.560 | 0.701 |
| g_w2 | =~ | blkdes_w2 | 0.619 | 0.036 | 17.366 | 0.000 | 0.549 | 0.689 |
| g_w2 | =~ | matreas_w2 | 0.558 | 0.039 | 14.311 | 0.000 | 0.481 | 0.634 |
| g_w2 | =~ | spantot_w2 | 0.475 | 0.043 | 11.031 | 0.000 | 0.391 | 0.559 |
| g_w2 | =~ | digsym_w2 | 0.683 | 0.032 | 21.217 | 0.000 | 0.620 | 0.746 |
| g_w2 | =~ | symsear_w2 | 0.667 | 0.033 | 19.950 | 0.000 | 0.601 | 0.732 |
| g_w2 | =~ | crtmean_invsq_w2 | 0.517 | 0.041 | 12.490 | 0.000 | 0.436 | 0.598 |
| g_w2 | =~ | ittotal_squared_w2 | 0.469 | 0.043 | 10.913 | 0.000 | 0.385 | 0.553 |
| g_w2 | =~ | vpatotal_w2 | 0.416 | 0.047 | 8.885 | 0.000 | 0.325 | 0.508 |
| g_w2 | =~ | lmtotal_w2 | 0.479 | 0.043 | 11.071 | 0.000 | 0.394 | 0.563 |
| g_w2 | =~ | digback_w2 | 0.499 | 0.042 | 12.014 | 0.000 | 0.418 | 0.581 |
| g_w2 | =~ | vftot_w2 | 0.511 | 0.041 | 12.474 | 0.000 | 0.431 | 0.592 |
| g_w2 | =~ | nart_w2 | 0.624 | 0.036 | 17.394 | 0.000 | 0.554 | 0.695 |
| g_w2 | =~ | wtar_w2 | 0.636 | 0.035 | 18.007 | 0.000 | 0.566 | 0.705 |
| g_w3 | =~ | blkdes_w3 | 0.677 | 0.032 | 21.426 | 0.000 | 0.615 | 0.739 |
| g_w3 | =~ | matreas_w3 | 0.605 | 0.036 | 16.764 | 0.000 | 0.534 | 0.676 |
| g_w3 | =~ | spantot_w3 | 0.484 | 0.042 | 11.563 | 0.000 | 0.402 | 0.566 |
| g_w3 | =~ | digsym_w3 | 0.713 | 0.030 | 23.808 | 0.000 | 0.654 | 0.771 |
| g_w3 | =~ | symsear_w3 | 0.710 | 0.030 | 23.680 | 0.000 | 0.651 | 0.769 |
| g_w3 | =~ | crtmean_invsq_w3 | 0.533 | 0.040 | 13.234 | 0.000 | 0.454 | 0.612 |
| g_w3 | =~ | ittotal_squared_w3 | 0.472 | 0.044 | 10.843 | 0.000 | 0.387 | 0.558 |
| g_w3 | =~ | vpatotal_w3 | 0.438 | 0.046 | 9.487 | 0.000 | 0.348 | 0.529 |
| g_w3 | =~ | lmtotal_w3 | 0.522 | 0.040 | 12.897 | 0.000 | 0.442 | 0.601 |
| g_w3 | =~ | digback_w3 | 0.503 | 0.041 | 12.264 | 0.000 | 0.422 | 0.583 |
| g_w3 | =~ | vftot_w3 | 0.475 | 0.042 | 11.215 | 0.000 | 0.392 | 0.558 |
| g_w3 | =~ | nart_total_w3 | 0.593 | 0.037 | 16.140 | 0.000 | 0.521 | 0.665 |
| g_w3 | =~ | wtar_total_w3 | 0.564 | 0.038 | 14.708 | 0.000 | 0.489 | 0.640 |
| g_w4 | =~ | blkdes_w4 | 0.615 | 0.035 | 17.560 | 0.000 | 0.547 | 0.684 |
| g_w4 | =~ | matreas_w4 | 0.633 | 0.034 | 18.585 | 0.000 | 0.567 | 0.700 |
| g_w4 | =~ | spantot_w4 | 0.499 | 0.041 | 12.229 | 0.000 | 0.419 | 0.580 |
| g_w4 | =~ | digsym_w4 | 0.724 | 0.029 | 25.205 | 0.000 | 0.668 | 0.780 |
| g_w4 | =~ | symsear_w4 | 0.725 | 0.028 | 25.612 | 0.000 | 0.669 | 0.780 |
| g_w4 | =~ | crtmean_invsq_w4 | 0.540 | 0.039 | 13.754 | 0.000 | 0.463 | 0.616 |
| g_w4 | =~ | ittotal_squared_w4 | 0.488 | 0.045 | 10.766 | 0.000 | 0.399 | 0.577 |
| g_w4 | =~ | vpatotal_w4 | 0.502 | 0.044 | 11.462 | 0.000 | 0.416 | 0.588 |
| g_w4 | =~ | lmtotal_w4 | 0.569 | 0.038 | 15.141 | 0.000 | 0.495 | 0.643 |
| g_w4 | =~ | digback_w4 | 0.552 | 0.038 | 14.434 | 0.000 | 0.477 | 0.627 |
| g_w4 | =~ | vftot_w4 | 0.504 | 0.041 | 12.407 | 0.000 | 0.424 | 0.583 |
| g_w4 | =~ | nart_total_w4 | 0.587 | 0.037 | 15.996 | 0.000 | 0.515 | 0.658 |
| g_w4 | =~ | wtar_total_w4 | 0.568 | 0.038 | 15.075 | 0.000 | 0.494 | 0.642 |
| g_w5 | =~ | blkdes_w5 | 0.650 | 0.032 | 20.177 | 0.000 | 0.587 | 0.713 |
| g_w5 | =~ | matreas_w5 | 0.626 | 0.034 | 18.276 | 0.000 | 0.559 | 0.693 |
| g_w5 | =~ | spantot_w5 | 0.581 | 0.036 | 16.099 | 0.000 | 0.510 | 0.652 |
| g_w5 | =~ | digsym_w5 | 0.805 | 0.022 | 36.972 | 0.000 | 0.763 | 0.848 |
| g_w5 | =~ | symsear_w5 | 0.807 | 0.022 | 36.870 | 0.000 | 0.764 | 0.850 |
| g_w5 | =~ | crtmean_invsq_w5 | 0.681 | 0.030 | 22.489 | 0.000 | 0.621 | 0.740 |
| g_w5 | =~ | ittotal_squared_w5 | 0.598 | 0.039 | 15.334 | 0.000 | 0.522 | 0.675 |
| g_w5 | =~ | vpa_total_w5 | 0.545 | 0.043 | 12.687 | 0.000 | 0.461 | 0.629 |
| g_w5 | =~ | lmtotal_w5 | 0.624 | 0.034 | 18.415 | 0.000 | 0.557 | 0.690 |
| g_w5 | =~ | digback_w5 | 0.515 | 0.040 | 12.986 | 0.000 | 0.437 | 0.592 |
| g_w5 | =~ | vftot_w5 | 0.516 | 0.040 | 12.960 | 0.000 | 0.438 | 0.594 |
| g_w5 | =~ | nart_total_w5 | 0.528 | 0.039 | 13.393 | 0.000 | 0.451 | 0.606 |
| g_w5 | =~ | wtar_total_w5 | 0.519 | 0.040 | 13.019 | 0.000 | 0.441 | 0.598 |
| nart_w1 | ~~ | wtar_w1 | 0.815 | 0.018 | 44.921 | 0.000 | 0.780 | 0.851 |
| nart_w2 | ~~ | wtar_w2 | 0.829 | 0.017 | 49.473 | 0.000 | 0.797 | 0.862 |
| nart_total_w3 | ~~ | wtar_total_w3 | 0.826 | 0.016 | 50.215 | 0.000 | 0.794 | 0.859 |
| nart_total_w4 | ~~ | wtar_total_w4 | 0.832 | 0.016 | 52.298 | 0.000 | 0.801 | 0.863 |
| nart_total_w5 | ~~ | wtar_total_w5 | 0.852 | 0.014 | 61.294 | 0.000 | 0.825 | 0.879 |

*Supplementary Table 8. Standardized solution of g model, model loadings: Model B1, B2, B3, B4, and B5 (separate models for each wave)*

| **lhs** | **op** | **rhs** | **est.std** | **se** | **z** | **pvalue** | **ci.lower** | **ci.upper** |
| --- | --- | --- | --- | --- | --- | --- | --- | --- |
| g_w1 | =~ | blkdes_w1 | 0.633 | 0.036 | 17.781 | 0.000 | 0.563 | 0.703 |
| g_w1 | =~ | matreas_w1 | 0.603 | 0.037 | 16.127 | 0.000 | 0.530 | 0.676 |
| g_w1 | =~ | spantot_w1 | 0.452 | 0.044 | 10.220 | 0.000 | 0.365 | 0.538 |
| g_w1 | =~ | digsym_w1 | 0.626 | 0.037 | 16.967 | 0.000 | 0.553 | 0.698 |
| g_w1 | =~ | symsear_w1 | 0.681 | 0.034 | 20.184 | 0.000 | 0.614 | 0.747 |
| g_w1 | =~ | crtmean_invsq_w1 | 0.429 | 0.046 | 9.321 | 0.000 | 0.339 | 0.520 |
| g_w1 | =~ | ittotal_squared_w1 | 0.268 | 0.051 | 5.277 | 0.000 | 0.168 | 0.367 |
| g_w1 | =~ | vpatotal_w1 | 0.394 | 0.047 | 8.301 | 0.000 | 0.301 | 0.487 |
| g_w1 | =~ | lmtotal_w1 | 0.444 | 0.045 | 9.837 | 0.000 | 0.355 | 0.532 |
| g_w1 | =~ | digback_w1 | 0.508 | 0.042 | 12.219 | 0.000 | 0.426 | 0.589 |
| g_w1 | =~ | vftot_w1 | 0.494 | 0.042 | 11.693 | 0.000 | 0.411 | 0.577 |
| g_w1 | =~ | nart_w1 | 0.627 | 0.036 | 17.309 | 0.000 | 0.556 | 0.698 |
| g_w1 | =~ | wtar_w1 | 0.630 | 0.036 | 17.494 | 0.000 | 0.560 | 0.701 |
| nart_w1 | ~~ | wtar_w1 | 0.815 | 0.018 | 44.921 | 0.000 | 0.780 | 0.851 |
| g_w2 | =~ | blkdes_w2 | 0.619 | 0.036 | 17.367 | 0.000 | 0.549 | 0.689 |
| g_w2 | =~ | matreas_w2 | 0.558 | 0.039 | 14.311 | 0.000 | 0.481 | 0.634 |
| g_w2 | =~ | spantot_w2 | 0.475 | 0.043 | 11.031 | 0.000 | 0.391 | 0.559 |
| g_w2 | =~ | digsym_w2 | 0.683 | 0.032 | 21.217 | 0.000 | 0.620 | 0.746 |
| g_w2 | =~ | symsear_w2 | 0.667 | 0.033 | 19.950 | 0.000 | 0.601 | 0.732 |
| g_w2 | =~ | crtmean_invsq_w2 | 0.517 | 0.041 | 12.490 | 0.000 | 0.436 | 0.598 |
| g_w2 | =~ | ittotal_squared_w2 | 0.469 | 0.043 | 10.913 | 0.000 | 0.385 | 0.553 |
| g_w2 | =~ | vpatotal_w2 | 0.416 | 0.047 | 8.885 | 0.000 | 0.325 | 0.508 |
| g_w2 | =~ | lmtotal_w2 | 0.479 | 0.043 | 11.071 | 0.000 | 0.394 | 0.563 |
| g_w2 | =~ | digback_w2 | 0.499 | 0.042 | 12.014 | 0.000 | 0.418 | 0.581 |
| g_w2 | =~ | vftot_w2 | 0.511 | 0.041 | 12.474 | 0.000 | 0.431 | 0.592 |
| g_w2 | =~ | nart_w2 | 0.624 | 0.036 | 17.394 | 0.000 | 0.554 | 0.695 |
| g_w2 | =~ | wtar_w2 | 0.636 | 0.035 | 18.007 | 0.000 | 0.566 | 0.705 |
| nart_w2 | ~~ | wtar_w2 | 0.829 | 0.017 | 49.474 | 0.000 | 0.797 | 0.862 |
| g_w3 | =~ | blkdes_w3 | 0.677 | 0.032 | 21.426 | 0.000 | 0.615 | 0.739 |
| g_w3 | =~ | matreas_w3 | 0.605 | 0.036 | 16.764 | 0.000 | 0.534 | 0.676 |
| g_w3 | =~ | spantot_w3 | 0.484 | 0.042 | 11.563 | 0.000 | 0.402 | 0.566 |
| g_w3 | =~ | digsym_w3 | 0.713 | 0.030 | 23.808 | 0.000 | 0.654 | 0.771 |
| g_w3 | =~ | symsear_w3 | 0.710 | 0.030 | 23.680 | 0.000 | 0.651 | 0.769 |
| g_w3 | =~ | crtmean_invsq_w3 | 0.533 | 0.040 | 13.234 | 0.000 | 0.454 | 0.612 |
| g_w3 | =~ | ittotal_squared_w3 | 0.472 | 0.044 | 10.843 | 0.000 | 0.387 | 0.558 |
| g_w3 | =~ | vpatotal_w3 | 0.438 | 0.046 | 9.487 | 0.000 | 0.348 | 0.529 |
| g_w3 | =~ | lmtotal_w3 | 0.522 | 0.040 | 12.897 | 0.000 | 0.442 | 0.601 |
| g_w3 | =~ | digback_w3 | 0.503 | 0.041 | 12.264 | 0.000 | 0.422 | 0.583 |
| g_w3 | =~ | vftot_w3 | 0.475 | 0.042 | 11.215 | 0.000 | 0.392 | 0.558 |
| g_w3 | =~ | nart_total_w3 | 0.593 | 0.037 | 16.140 | 0.000 | 0.521 | 0.665 |
| g_w3 | =~ | wtar_total_w3 | 0.564 | 0.038 | 14.708 | 0.000 | 0.489 | 0.640 |
| nart_total_w3 | ~~ | wtar_total_w3 | 0.826 | 0.016 | 50.215 | 0.000 | 0.794 | 0.859 |
| g_w4 | =~ | blkdes_w4 | 0.615 | 0.035 | 17.558 | 0.000 | 0.547 | 0.684 |
| g_w4 | =~ | matreas_w4 | 0.633 | 0.034 | 18.585 | 0.000 | 0.567 | 0.700 |
| g_w4 | =~ | spantot_w4 | 0.499 | 0.041 | 12.229 | 0.000 | 0.419 | 0.580 |
| g_w4 | =~ | digsym_w4 | 0.724 | 0.029 | 25.205 | 0.000 | 0.668 | 0.780 |
| g_w4 | =~ | symsear_w4 | 0.725 | 0.028 | 25.612 | 0.000 | 0.669 | 0.780 |
| g_w4 | =~ | crtmean_invsq_w4 | 0.540 | 0.039 | 13.754 | 0.000 | 0.463 | 0.617 |
| g_w4 | =~ | ittotal_squared_w4 | 0.488 | 0.045 | 10.766 | 0.000 | 0.399 | 0.577 |
| g_w4 | =~ | vpatotal_w4 | 0.502 | 0.044 | 11.462 | 0.000 | 0.416 | 0.588 |
| g_w4 | =~ | lmtotal_w4 | 0.569 | 0.038 | 15.141 | 0.000 | 0.495 | 0.643 |
| g_w4 | =~ | digback_w4 | 0.552 | 0.038 | 14.434 | 0.000 | 0.477 | 0.627 |
| g_w4 | =~ | vftot_w4 | 0.504 | 0.041 | 12.407 | 0.000 | 0.424 | 0.583 |
| g_w4 | =~ | nart_total_w4 | 0.587 | 0.037 | 15.996 | 0.000 | 0.515 | 0.658 |
| g_w4 | =~ | wtar_total_w4 | 0.568 | 0.038 | 15.075 | 0.000 | 0.494 | 0.642 |
| nart_total_w4 | ~~ | wtar_total_w4 | 0.832 | 0.016 | 52.298 | 0.000 | 0.801 | 0.863 |
| g_w5 | =~ | blkdes_w5 | 0.650 | 0.032 | 20.179 | 0.000 | 0.587 | 0.713 |
| g_w5 | =~ | matreas_w5 | 0.626 | 0.034 | 18.276 | 0.000 | 0.559 | 0.693 |
| g_w5 | =~ | spantot_w5 | 0.581 | 0.036 | 16.099 | 0.000 | 0.510 | 0.652 |
| g_w5 | =~ | digsym_w5 | 0.805 | 0.022 | 36.972 | 0.000 | 0.763 | 0.848 |
| g_w5 | =~ | symsear_w5 | 0.807 | 0.022 | 36.871 | 0.000 | 0.764 | 0.850 |
| g_w5 | =~ | crtmean_invsq_w5 | 0.681 | 0.030 | 22.489 | 0.000 | 0.621 | 0.740 |
| g_w5 | =~ | ittotal_squared_w5 | 0.598 | 0.039 | 15.334 | 0.000 | 0.522 | 0.675 |
| g_w5 | =~ | vpa_total_w5 | 0.545 | 0.043 | 12.688 | 0.000 | 0.461 | 0.629 |
| g_w5 | =~ | lmtotal_w5 | 0.624 | 0.034 | 18.415 | 0.000 | 0.557 | 0.690 |
| g_w5 | =~ | digback_w5 | 0.515 | 0.040 | 12.986 | 0.000 | 0.437 | 0.592 |
| g_w5 | =~ | vftot_w5 | 0.516 | 0.040 | 12.960 | 0.000 | 0.438 | 0.594 |
| g_w5 | =~ | nart_total_w5 | 0.528 | 0.039 | 13.393 | 0.000 | 0.451 | 0.606 |
| g_w5 | =~ | wtar_total_w5 | 0.519 | 0.040 | 13.019 | 0.000 | 0.441 | 0.598 |
| nart_total_w5 | ~~ | wtar_total_w5 | 0.852 | 0.014 | 61.294 | 0.000 | 0.825 | 0.879 |

*Supplementary Table 9. Standardized solution of hierarchical g model, model loadings*

| **lhs** | **op** | **rhs** | **est.std** | **se** | **z** | **pvalue** | **ci.lower** | **ci.upper** |
| --- | --- | --- | --- | --- | --- | --- | --- | --- |
| Vis_w1 | =~ | blkdes_w1 | 0.750 | 0.035 | 21.416 | 0.000 | 0.681 | 0.819 |
| Vis_w1 | =~ | matreas_w1 | 0.725 | 0.035 | 20.502 | 0.000 | 0.656 | 0.794 |
| Vis_w1 | =~ | spantot_w1 | 0.461 | 0.046 | 9.938 | 0.000 | 0.370 | 0.552 |
| Speed_w1 | =~ | digsym_w1 | 0.773 | 0.033 | 23.199 | 0.000 | 0.708 | 0.838 |
| Speed_w1 | =~ | symsear_w1 | 0.776 | 0.033 | 23.241 | 0.000 | 0.710 | 0.841 |
| Speed_w1 | =~ | crtmean_invsq_w1 | 0.544 | 0.042 | 13.031 | 0.000 | 0.462 | 0.626 |
| Speed_w1 | =~ | ittotal_squared_w1 | 0.320 | 0.051 | 6.330 | 0.000 | 0.221 | 0.420 |
| Mem_w1 | =~ | vpatotal_w1 | 0.553 | 0.054 | 10.252 | 0.000 | 0.447 | 0.659 |
| Mem_w1 | =~ | lmtotal_w1 | 0.611 | 0.052 | 11.745 | 0.000 | 0.509 | 0.713 |
| Mem_w1 | =~ | digback_w1 | 0.524 | 0.049 | 10.613 | 0.000 | 0.427 | 0.621 |
| Cryst_w1 | =~ | vftot_w1 | 0.468 | 0.040 | 11.781 | 0.000 | 0.390 | 0.546 |
| Cryst_w1 | =~ | nart_w1 | 0.937 | 0.013 | 71.931 | 0.000 | 0.911 | 0.962 |
| Cryst_w1 | =~ | wtar_w1 | 0.947 | 0.013 | 74.185 | 0.000 | 0.922 | 0.972 |
| Vis_w2 | =~ | blkdes_w2 | 0.754 | 0.037 | 20.478 | 0.000 | 0.682 | 0.826 |
| Vis_w2 | =~ | matreas_w2 | 0.678 | 0.039 | 17.585 | 0.000 | 0.603 | 0.754 |
| Vis_w2 | =~ | spantot_w2 | 0.509 | 0.045 | 11.296 | 0.000 | 0.421 | 0.597 |
| Speed_w2 | =~ | digsym_w2 | 0.793 | 0.029 | 27.334 | 0.000 | 0.736 | 0.850 |
| Speed_w2 | =~ | symsear_w2 | 0.739 | 0.031 | 23.742 | 0.000 | 0.678 | 0.800 |
| Speed_w2 | =~ | crtmean_invsq_w2 | 0.603 | 0.038 | 15.911 | 0.000 | 0.529 | 0.678 |
| Speed_w2 | =~ | ittotal_squared_w2 | 0.484 | 0.044 | 10.985 | 0.000 | 0.397 | 0.570 |
| Mem_w2 | =~ | vpatotal_w2 | 0.604 | 0.047 | 12.776 | 0.000 | 0.511 | 0.697 |
| Mem_w2 | =~ | lmtotal_w2 | 0.680 | 0.045 | 15.047 | 0.000 | 0.592 | 0.769 |
| Mem_w2 | =~ | digback_w2 | 0.499 | 0.050 | 9.964 | 0.000 | 0.401 | 0.597 |
| Cryst_w2 | =~ | vftot_w2 | 0.481 | 0.039 | 12.340 | 0.000 | 0.404 | 0.557 |
| Cryst_w2 | =~ | nart_w2 | 0.936 | 0.012 | 76.999 | 0.000 | 0.912 | 0.960 |
| Cryst_w2 | =~ | wtar_w2 | 0.957 | 0.012 | 82.377 | 0.000 | 0.934 | 0.980 |
| Vis_w3 | =~ | blkdes_w3 | 0.768 | 0.032 | 23.827 | 0.000 | 0.705 | 0.831 |
| Vis_w3 | =~ | matreas_w3 | 0.718 | 0.034 | 20.965 | 0.000 | 0.651 | 0.785 |
| Vis_w3 | =~ | spantot_w3 | 0.519 | 0.043 | 12.084 | 0.000 | 0.435 | 0.603 |
| Speed_w3 | =~ | digsym_w3 | 0.784 | 0.029 | 27.493 | 0.000 | 0.728 | 0.840 |
| Speed_w3 | =~ | symsear_w3 | 0.781 | 0.028 | 27.465 | 0.000 | 0.725 | 0.836 |
| Speed_w3 | =~ | crtmean_invsq_w3 | 0.618 | 0.037 | 16.803 | 0.000 | 0.546 | 0.690 |
| Speed_w3 | =~ | ittotal_squared_w3 | 0.492 | 0.044 | 11.178 | 0.000 | 0.406 | 0.578 |
| Mem_w3 | =~ | vpatotal_w3 | 0.581 | 0.049 | 11.782 | 0.000 | 0.484 | 0.678 |
| Mem_w3 | =~ | lmtotal_w3 | 0.664 | 0.045 | 14.815 | 0.000 | 0.576 | 0.752 |
| Mem_w3 | =~ | digback_w3 | 0.530 | 0.047 | 11.316 | 0.000 | 0.438 | 0.622 |
| Cryst_w3 | =~ | vftot_w3 | 0.472 | 0.040 | 11.874 | 0.000 | 0.394 | 0.549 |
| Cryst_w3 | =~ | nart_total_w3 | 0.954 | 0.013 | 71.390 | 0.000 | 0.928 | 0.980 |
| Cryst_w3 | =~ | wtar_total_w3 | 0.926 | 0.014 | 66.152 | 0.000 | 0.898 | 0.953 |
| Vis_w4 | =~ | blkdes_w4 | 0.695 | 0.035 | 19.759 | 0.000 | 0.626 | 0.764 |
| Vis_w4 | =~ | matreas_w4 | 0.765 | 0.033 | 23.401 | 0.000 | 0.701 | 0.829 |
| Vis_w4 | =~ | spantot_w4 | 0.530 | 0.043 | 12.374 | 0.000 | 0.446 | 0.613 |
| Speed_w4 | =~ | digsym_w4 | 0.806 | 0.027 | 29.513 | 0.000 | 0.752 | 0.859 |
| Speed_w4 | =~ | symsear_w4 | 0.757 | 0.029 | 25.713 | 0.000 | 0.699 | 0.814 |
| Speed_w4 | =~ | crtmean_invsq_w4 | 0.599 | 0.038 | 15.808 | 0.000 | 0.524 | 0.673 |
| Speed_w4 | =~ | ittotal_squared_w4 | 0.526 | 0.044 | 11.828 | 0.000 | 0.439 | 0.614 |
| Mem_w4 | =~ | vpatotal_w4 | 0.632 | 0.045 | 14.052 | 0.000 | 0.544 | 0.720 |
| Mem_w4 | =~ | lmtotal_w4 | 0.684 | 0.041 | 16.806 | 0.000 | 0.604 | 0.764 |
| Mem_w4 | =~ | digback_w4 | 0.571 | 0.043 | 13.271 | 0.000 | 0.487 | 0.655 |
| Cryst_w4 | =~ | wtar_total_w4 | 0.935 | 0.014 | 68.449 | 0.000 | 0.908 | 0.962 |
| Cryst_w4 | =~ | vftot_w4 | 0.420 | 0.042 | 9.985 | 0.000 | 0.338 | 0.503 |
| Cryst_w4 | =~ | nart_total_w4 | 0.948 | 0.013 | 70.813 | 0.000 | 0.922 | 0.974 |
| Vis_w5 | =~ | blkdes_w5 | 0.729 | 0.032 | 22.574 | 0.000 | 0.666 | 0.792 |
| Vis_w5 | =~ | matreas_w5 | 0.730 | 0.032 | 22.617 | 0.000 | 0.667 | 0.794 |
| Vis_w5 | =~ | spantot_w5 | 0.606 | 0.038 | 15.881 | 0.000 | 0.531 | 0.681 |
| Speed_w5 | =~ | digsym_w5 | 0.861 | 0.019 | 44.996 | 0.000 | 0.823 | 0.898 |
| Speed_w5 | =~ | symsear_w5 | 0.837 | 0.021 | 40.442 | 0.000 | 0.797 | 0.878 |
| Speed_w5 | =~ | crtmean_invsq_w5 | 0.694 | 0.030 | 23.113 | 0.000 | 0.635 | 0.753 |
| Speed_w5 | =~ | ittotal_squared_w5 | 0.614 | 0.038 | 16.101 | 0.000 | 0.540 | 0.689 |
| Mem_w5 | =~ | vpa_total_w5 | 0.701 | 0.041 | 17.009 | 0.000 | 0.621 | 0.782 |
| Mem_w5 | =~ | lmtotal_w5 | 0.760 | 0.036 | 21.214 | 0.000 | 0.689 | 0.830 |
| Mem_w5 | =~ | digback_w5 | 0.537 | 0.045 | 11.857 | 0.000 | 0.448 | 0.626 |
| Cryst_w5 | =~ | vftot_w5 | 0.456 | 0.041 | 11.170 | 0.000 | 0.376 | 0.536 |
| Cryst_w5 | =~ | nart_total_w5 | 0.948 | 0.013 | 70.659 | 0.000 | 0.922 | 0.975 |
| Cryst_w5 | =~ | wtar_total_w5 | 0.940 | 0.014 | 69.184 | 0.000 | 0.914 | 0.967 |
| g_w1 | =~ | Vis_w1 | 0.781 | 0.046 | 17.045 | 0.000 | 0.691 | 0.871 |
| g_w1 | =~ | Speed_w1 | 0.644 | 0.048 | 13.352 | 0.000 | 0.550 | 0.739 |
| g_w1 | =~ | Mem_w1 | 0.883 | 0.060 | 14.731 | 0.000 | 0.766 | 1.001 |
| g_w1 | =~ | Cryst_w1 | 0.760 | 0.037 | 20.493 | 0.000 | 0.687 | 0.832 |
| g_w2 | =~ | Vis_w2 | 0.762 | 0.047 | 16.091 | 0.000 | 0.669 | 0.854 |
| g_w2 | =~ | Speed_w2 | 0.696 | 0.046 | 15.273 | 0.000 | 0.607 | 0.785 |
| g_w2 | =~ | Mem_w2 | 0.843 | 0.054 | 15.729 | 0.000 | 0.738 | 0.948 |
| g_w2 | =~ | Cryst_w2 | 0.770 | 0.037 | 20.991 | 0.000 | 0.698 | 0.842 |
| g_w3 | =~ | Vis_w3 | 0.831 | 0.041 | 20.322 | 0.000 | 0.751 | 0.911 |
| g_w3 | =~ | Speed_w3 | 0.767 | 0.041 | 18.873 | 0.000 | 0.687 | 0.847 |
| g_w3 | =~ | Mem_w3 | 0.877 | 0.053 | 16.466 | 0.000 | 0.773 | 0.982 |
| g_w3 | =~ | Cryst_w3 | 0.691 | 0.039 | 17.629 | 0.000 | 0.614 | 0.768 |
| g_w4 | =~ | Vis_w4 | 0.834 | 0.039 | 21.331 | 0.000 | 0.757 | 0.911 |
| g_w4 | =~ | Speed_w4 | 0.796 | 0.038 | 20.958 | 0.000 | 0.721 | 0.870 |
| g_w4 | =~ | Mem_w4 | 0.896 | 0.046 | 19.626 | 0.000 | 0.806 | 0.985 |
| g_w4 | =~ | Cryst_w4 | 0.681 | 0.038 | 18.130 | 0.000 | 0.607 | 0.754 |
| g_w5 | =~ | Vis_w5 | 0.904 | 0.033 | 27.778 | 0.000 | 0.841 | 0.968 |
| g_w5 | =~ | Speed_w5 | 0.833 | 0.030 | 27.321 | 0.000 | 0.773 | 0.893 |
| g_w5 | =~ | Mem_w5 | 0.868 | 0.041 | 20.966 | 0.000 | 0.787 | 0.949 |
| g_w5 | =~ | Cryst_w5 | 0.624 | 0.040 | 15.494 | 0.000 | 0.545 | 0.703 |

*Supplementary Table 10. Proportion of variances explained by each domain in the hierarchical model, and their Pearson’s r correlations with the mean age at each wave.*

|  | **Wave** | | | | |  |
| --- | --- | --- | --- | --- | --- | --- |
| **Domain** | **1** | **2** | **3** | **4** | **5** | ***r* (*p*)** |
| Visuospatial | 43.36% | 42.92% | 45.81% | 44.95% | 47.74% | 0.877 (.051) |
| Processing Speed | 39.94% | 44.34% | 46.19% | 46.43% | 57.52% | 0.889 (.044) |
| Verbal Memory | 31.80% | 35.89% | 35.33% | 39.78% | 45.24% | 0.993 (.021) |
| Crystallised | 66.45% | 67.46% | 66.30% | 64.98% | 66.38% | -0.502 (.389) |

*Supplementary Table 11. Correlations between changes across waves in domain scores across waves (extracted from the hierarchical model): Pearson’s r (p value, FDR Q).*

| **Domain** | **Visuospatial** | **Processing Speed** | **Verbal Memory** |
| --- | --- | --- | --- |
| Visuospatial | 1.000 |  |  |
| Processing Speed | 0.990 (.001, .006) | 1.000 |  |
| Verbal Memory | 0.777 (.122, .146) | 0.951 (.013, .026) | 1.000 |
| Crystallised | -0.877 (.051, .077) | -0.338 (.578, .578) | -0.974 (.005, .015) |

*Supplementary Table 12. Correlations between domain scores across waves (extracted from the hierarchical model): Pearson’s r. The correlations between changes in these across waves are shown in Supplementary Table 11.*

|  | **Vis_w1** | **Speed_w1** | **Mem_w1** | **Cryst_w1** | **g_w1** | **Vis_w2** | **Speed_w2** | **Mem_w2** | **Cryst_w2** | **g_w2** | **Vis_w3** | **Speed_w3** | **Mem_w3** | **Cryst_w3** | **g_w3** | **Vis_w4** | **Speed_w4** | **Mem_w4** | **Cryst_w4** | **g_w4** | **Vis_w5** | **Speed_w5** | **Mem_w5** | **Cryst_w5** | **g_w5** |
| --- | --- | --- | --- | --- | --- | --- | --- | --- | --- | --- | --- | --- | --- | --- | --- | --- | --- | --- | --- | --- | --- | --- | --- | --- | --- |
| **Vis_w1** | 1.00 | 0.67 | 0.83 | 0.66 | 0.90 | 0.86 | 0.66 | 0.73 | 0.65 | 0.82 | 0.86 | 0.68 | 0.76 | 0.63 | 0.83 | 0.85 | 0.65 | 0.73 | 0.63 | 0.79 | 0.77 | 0.57 | 0.66 | 0.63 | 0.73 |
| **Speed_w1** | 0.67 | 1.00 | 0.69 | 0.54 | 0.76 | 0.64 | 0.85 | 0.61 | 0.54 | 0.72 | 0.65 | 0.82 | 0.65 | 0.53 | 0.73 | 0.68 | 0.80 | 0.66 | 0.52 | 0.73 | 0.67 | 0.73 | 0.63 | 0.55 | 0.71 |
| **Mem_w1** | 0.83 | 0.69 | 1.00 | 0.81 | 0.97 | 0.74 | 0.66 | 0.86 | 0.77 | 0.87 | 0.75 | 0.66 | 0.84 | 0.77 | 0.85 | 0.76 | 0.64 | 0.81 | 0.75 | 0.82 | 0.71 | 0.56 | 0.73 | 0.76 | 0.74 |
| **Cryst_w1** | 0.66 | 0.54 | 0.81 | 1.00 | 0.87 | 0.63 | 0.55 | 0.75 | 0.95 | 0.83 | 0.63 | 0.54 | 0.71 | 0.95 | 0.76 | 0.63 | 0.53 | 0.70 | 0.93 | 0.74 | 0.59 | 0.45 | 0.62 | 0.94 | 0.64 |
| **g_w1** | 0.90 | 0.76 | 0.97 | 0.87 | 1.00 | 0.81 | 0.73 | 0.86 | 0.83 | 0.92 | 0.81 | 0.72 | 0.85 | 0.83 | 0.89 | 0.82 | 0.70 | 0.83 | 0.81 | 0.86 | 0.76 | 0.62 | 0.75 | 0.82 | 0.79 |
| **Vis_w2** | 0.86 | 0.64 | 0.74 | 0.63 | 0.81 | 1.00 | 0.71 | 0.78 | 0.66 | 0.88 | 0.87 | 0.71 | 0.76 | 0.62 | 0.84 | 0.87 | 0.68 | 0.75 | 0.64 | 0.81 | 0.81 | 0.62 | 0.69 | 0.64 | 0.77 |
| **Speed_w2** | 0.66 | 0.85 | 0.66 | 0.55 | 0.73 | 0.71 | 1.00 | 0.70 | 0.58 | 0.81 | 0.71 | 0.87 | 0.71 | 0.58 | 0.79 | 0.72 | 0.85 | 0.71 | 0.57 | 0.79 | 0.74 | 0.80 | 0.69 | 0.59 | 0.78 |
| **Mem_w2** | 0.73 | 0.61 | 0.86 | 0.75 | 0.86 | 0.78 | 0.70 | 1.00 | 0.79 | 0.95 | 0.73 | 0.68 | 0.88 | 0.77 | 0.86 | 0.75 | 0.67 | 0.85 | 0.76 | 0.84 | 0.72 | 0.60 | 0.78 | 0.76 | 0.77 |
| **Cryst_w2** | 0.65 | 0.54 | 0.77 | 0.95 | 0.83 | 0.66 | 0.58 | 0.79 | 1.00 | 0.87 | 0.63 | 0.56 | 0.73 | 0.94 | 0.77 | 0.64 | 0.56 | 0.71 | 0.94 | 0.75 | 0.60 | 0.48 | 0.63 | 0.95 | 0.66 |
| **g_w2** | 0.82 | 0.72 | 0.87 | 0.83 | 0.92 | 0.88 | 0.81 | 0.95 | 0.87 | 1.00 | 0.82 | 0.77 | 0.88 | 0.84 | 0.92 | 0.84 | 0.76 | 0.86 | 0.84 | 0.90 | 0.80 | 0.68 | 0.79 | 0.85 | 0.83 |
| **Vis_w3** | 0.86 | 0.65 | 0.75 | 0.63 | 0.81 | 0.87 | 0.71 | 0.73 | 0.63 | 0.82 | 1.00 | 0.79 | 0.86 | 0.64 | 0.93 | 0.88 | 0.75 | 0.80 | 0.64 | 0.86 | 0.86 | 0.68 | 0.76 | 0.64 | 0.83 |
| **Speed_w3** | 0.68 | 0.82 | 0.66 | 0.54 | 0.72 | 0.71 | 0.87 | 0.68 | 0.56 | 0.77 | 0.79 | 1.00 | 0.80 | 0.56 | 0.87 | 0.77 | 0.90 | 0.78 | 0.55 | 0.84 | 0.81 | 0.86 | 0.76 | 0.57 | 0.84 |
| **Mem_w3** | 0.76 | 0.65 | 0.84 | 0.71 | 0.85 | 0.76 | 0.71 | 0.88 | 0.73 | 0.88 | 0.86 | 0.80 | 1.00 | 0.74 | 0.97 | 0.82 | 0.76 | 0.91 | 0.73 | 0.91 | 0.82 | 0.71 | 0.86 | 0.74 | 0.86 |
| **Cryst_w3** | 0.63 | 0.53 | 0.77 | 0.95 | 0.83 | 0.62 | 0.58 | 0.77 | 0.94 | 0.84 | 0.64 | 0.56 | 0.74 | 1.00 | 0.78 | 0.63 | 0.56 | 0.73 | 0.95 | 0.76 | 0.61 | 0.49 | 0.65 | 0.95 | 0.67 |
| **g_w3** | 0.83 | 0.73 | 0.85 | 0.76 | 0.89 | 0.84 | 0.79 | 0.86 | 0.77 | 0.92 | 0.93 | 0.87 | 0.97 | 0.78 | 1.00 | 0.88 | 0.83 | 0.91 | 0.77 | 0.94 | 0.87 | 0.76 | 0.86 | 0.78 | 0.90 |
| **Vis_w4** | 0.85 | 0.68 | 0.76 | 0.63 | 0.82 | 0.87 | 0.72 | 0.75 | 0.64 | 0.84 | 0.88 | 0.77 | 0.82 | 0.63 | 0.88 | 1.00 | 0.81 | 0.87 | 0.64 | 0.93 | 0.89 | 0.73 | 0.79 | 0.66 | 0.86 |
| **Speed_w4** | 0.65 | 0.80 | 0.64 | 0.53 | 0.70 | 0.68 | 0.85 | 0.67 | 0.56 | 0.76 | 0.75 | 0.90 | 0.76 | 0.56 | 0.83 | 0.81 | 1.00 | 0.83 | 0.57 | 0.90 | 0.83 | 0.89 | 0.80 | 0.58 | 0.87 |
| **Mem_w4** | 0.73 | 0.66 | 0.81 | 0.70 | 0.83 | 0.75 | 0.71 | 0.85 | 0.71 | 0.86 | 0.80 | 0.78 | 0.91 | 0.73 | 0.91 | 0.87 | 0.83 | 1.00 | 0.73 | 0.97 | 0.85 | 0.75 | 0.91 | 0.73 | 0.89 |
| **Cryst_w4** | 0.63 | 0.52 | 0.75 | 0.93 | 0.81 | 0.64 | 0.57 | 0.76 | 0.94 | 0.84 | 0.64 | 0.55 | 0.73 | 0.95 | 0.77 | 0.64 | 0.57 | 0.73 | 1.00 | 0.77 | 0.62 | 0.49 | 0.65 | 0.94 | 0.67 |
| **g_w4** | 0.79 | 0.73 | 0.82 | 0.74 | 0.86 | 0.81 | 0.79 | 0.84 | 0.75 | 0.90 | 0.86 | 0.84 | 0.91 | 0.76 | 0.94 | 0.93 | 0.90 | 0.97 | 0.77 | 1.00 | 0.90 | 0.80 | 0.89 | 0.77 | 0.93 |
| **Vis_w5** | 0.77 | 0.67 | 0.71 | 0.59 | 0.76 | 0.81 | 0.74 | 0.72 | 0.60 | 0.80 | 0.86 | 0.81 | 0.82 | 0.61 | 0.87 | 0.89 | 0.83 | 0.85 | 0.62 | 0.90 | 1.00 | 0.87 | 0.89 | 0.64 | 0.97 |
| **Speed_w5** | 0.57 | 0.73 | 0.56 | 0.45 | 0.62 | 0.62 | 0.80 | 0.60 | 0.48 | 0.68 | 0.68 | 0.86 | 0.71 | 0.49 | 0.76 | 0.73 | 0.89 | 0.75 | 0.49 | 0.80 | 0.87 | 1.00 | 0.83 | 0.52 | 0.92 |
| **Mem_w5** | 0.66 | 0.63 | 0.73 | 0.62 | 0.75 | 0.69 | 0.69 | 0.78 | 0.63 | 0.79 | 0.76 | 0.76 | 0.86 | 0.65 | 0.86 | 0.79 | 0.80 | 0.91 | 0.65 | 0.89 | 0.89 | 0.83 | 1.00 | 0.66 | 0.95 |
| **Cryst_w5** | 0.63 | 0.55 | 0.76 | 0.94 | 0.82 | 0.64 | 0.59 | 0.76 | 0.95 | 0.85 | 0.64 | 0.57 | 0.74 | 0.95 | 0.78 | 0.66 | 0.58 | 0.73 | 0.94 | 0.77 | 0.64 | 0.52 | 0.66 | 1.00 | 0.70 |
| **g_w5** | 0.73 | 0.71 | 0.74 | 0.64 | 0.79 | 0.77 | 0.78 | 0.77 | 0.66 | 0.83 | 0.83 | 0.84 | 0.86 | 0.67 | 0.90 | 0.86 | 0.87 | 0.89 | 0.67 | 0.93 | 0.97 | 0.92 | 0.95 | 0.70 | 1.00 |

**R code for all models**

R code for Model A (all waves)

library(lavaan)

model_A <- '

g_w1 =~ blkdes_w1 + matreas_w1 + spantot_w1 + digsym_w1 + symsear_w1 + crtmean_invsq_w1 + ittotal_squared_w1 + vpatotal_w1 + lmtotal_w1 + digback_w1 + vftot_w1 + nart_w1 + wtar_w1

g_w2 =~ blkdes_w2 + matreas_w2 + spantot_w2 + digsym_w2 + symsear_w2 + crtmean_invsq_w2 + ittotal_squared_w2 + vpatotal_w2 + lmtotal_w2 + digback_w2 + vftot_w2 + nart_w2 + wtar_w2

g_w3 =~ blkdes_w3 + matreas_w3 + spantot_w3 + digsym_w3 + symsear_w3 + crtmean_invsq_w3 + ittotal_squared_w3 + vpatotal_w3 + lmtotal_w3 + digback_w3 + vftot_w3 + nart_total_w3 + wtar_total_w3

g_w4 =~ blkdes_w4 + matreas_w4 + spantot_w4 + digsym_w4 + symsear_w4 + crtmean_invsq_w4 + ittotal_squared_w4 + vpatotal_w4 + lmtotal_w4 + digback_w4 + vftot_w4 + nart_total_w4 + wtar_total_w4

g_w5 =~ blkdes_w5 + matreas_w5 + spantot_w5 + digsym_w5 + symsear_w5 + crtmean_invsq_w5 + ittotal_squared_w5 + vpa_total_w5 + lmtotal_w5 + digback_w5 + vftot_w5 + nart_total_w5 + wtar_total_w5

nart_w1 ~~ wtar_w1

nart_w2 ~~ wtar_w2

nart_total_w3 ~~ wtar_total_w3

nart_total_w4 ~~ wtar_total_w4

nart_total_w5 ~~ wtar_total_w5

g_w1 ~~ 0*g_w2 # set covariances between gs to 0

g_w1 ~~ 0*g_w3

g_w1 ~~ 0*g_w4

g_w1 ~~ 0*g_w5

g_w2 ~~ 0*g_w3

g_w2 ~~ 0*g_w4

g_w2 ~~ 0*g_w5

g_w3 ~~ 0*g_w4

g_w3 ~~ 0*g_w5

g_w4 ~~ 0*g_w5

blkdes_w1~ 0*1 # set item intercepts to 0

matreas_w1~ 0*1

spantot_w1~ 0*1

digsym_w1~ 0*1

crtmean_invsq_w1~ 0*1

symsear_w1~ 0*1

ittotal_squared_w1~ 0*1

nart_w1~0*1

wtar_w1~0*1

vftot_w1~0*1

vpatotal_w1~0*1

lmtotal_w1~0*1

digback_w1~0*1

blkdes_w2~ 0*1

matreas_w2~ 0*1

spantot_w2~ 0*1

digsym_w2~ 0*1

crtmean_invsq_w2~ 0*1

symsear_w2~ 0*1

ittotal_squared_w2~ 0*1

nart_w2~0*1

wtar_w2~0*1

vftot_w2~0*1

vpatotal_w2~0*1

lmtotal_w2~0*1

digback_w2~0*1

blkdes_w3~ 0*1

matreas_w3~ 0*1

spantot_w3~ 0*1

digsym_w3~ 0*1

crtmean_invsq_w3~ 0*1

symsear_w3~ 0*1

ittotal_squared_w3~ 0*1

nart_total_w3~0*1

wtar_total_w3~0*1

vftot_w3~0*1

vpatotal_w3~0*1

lmtotal_w3~0*1

digback_w3~0*1

blkdes_w4~ 0*1

matreas_w4~ 0*1

spantot_w4~ 0*1

digsym_w4~ 0*1

crtmean_invsq_w4~ 0*1

symsear_w4~ 0*1

ittotal_squared_w4~ 0*1

nart_total_w4~0*1

wtar_total_w4~0*1

vftot_w4~0*1

vpatotal_w4~0*1

lmtotal_w4~0*1

digback_w4~0*1

blkdes_w5~ 0*1

matreas_w5~ 0*1

spantot_w5~ 0*1

digsym_w5~ 0*1

crtmean_invsq_w5~ 0*1

symsear_w5~ 0*1

ittotal_squared_w5~ 0*1

nart_total_w5~0*1

wtar_total_w5~0*1

vftot_w5~0*1

vpa_total_w5~0*1

lmtotal_w5~0*1

digback_w5~0*1

# calculate g intercepts

g_w1 ~ 1

g_w2 ~ 1

g_w3 ~ 1

g_w4 ~ 1

g_w5 ~ 1

'

fit_A <- sem(model = model_A, data, missing = "ml.x")

R code for Models A1, A2, A3, A4, A5 (separate models for each wave)

library(lavaan)

model_A1 <- '

g_w1 =~ blkdes_g_w1 =~ blkdes_w1 + matreas_w1 + spantot_w1 + digsym_w1 + symsear_w1 + crtmean_invsq_w1 + ittotal_squared_w1 + vpatotal_w1 + lmtotal_w1 + digback_w1 + vftot_w1 + nart_w1 + wtar_w1

nart_w1 ~~ wtar_w1

g_w1 ~ 1

blkdes_w1~ 0*1

matreas_w1~ 0*1

spantot_w1~ 0*1

digsym_w1~ 0*1

crtmean_invsq_w1~ 0*1

symsear_w1~ 0*1

ittotal_squared_w1~ 0*1

vpatotal_w1 ~ 0*1

lmtotal_w1 ~ 0*1

digback_w1 ~ 0*1‘

fit_A1 <- sem(model = model_A1, data, missing = "ml.x")

model_A2 <- '

g_w2 =~ blkdes_w2 + matreas_w2 + spantot_w2 + digsym_w2 + symsear_w2 + crtmean_invsq_w2 + ittotal_squared_w2 + vpatotal_w2 + lmtotal_w2 + digback_w2 + vftot_w2 + nart_w2 + wtar_w2

nart_w2 ~~ wtar_w2

g_w2 ~ 1

blkdes_w2~ 0*1

matreas_w2~ 0*1

spantot_w2~ 0*1

digsym_w2~ 0*1

crtmean_invsq_w2~ 0*1

symsear_w2~ 0*1

ittotal_squared_w2~ 0*1

vpatotal_w2 ~ 0*1

lmtotal_w2 ~ 0*1

digback_w2 ~ 0*1

‘

fit_A2 <- sem(model = model_A2,model_variables_scaled_completers, missing = "ml.x")

model_A3 <- '

g_w3 =~ blkdes_w3 + matreas_w3 + spantot_w3 + digsym_w3 + symsear_w3 + crtmean_invsq_w3 + ittotal_squared_w3 + vpatotal_w3 + lmtotal_w3 + digback_w3 + vftot_w3 + nart_total_w3 + wtar_total_w3

nart_total_w3 ~~ wtar_total_w3

g_w3 ~ 1

blkdes_w3~ 0*1

matreas_w3~ 0*1

spantot_w3~ 0*1

digsym_w3~ 0*1

crtmean_invsq_w3~ 0*1

symsear_w3~ 0*1

ittotal_squared_w3~ 0*1

vpatotal_w3 ~ 0*1

lmtotal_w3 ~ 0*1

digback_w3 ~ 0*1

‘

fit_A3 <- sem(model = model_A3, model_variables_scaled_completers, missing = "ml.x")

model_A4 <- '

g_w4 =~ blkdes_w4 + matreas_w4 + spantot_w4 + digsym_w4 + symsear_w4 + crtmean_invsq_w4 + ittotal_squared_w4 + vpatotal_w4 + lmtotal_w4 + digback_w4 + vftot_w4 + nart_total_w4 + wtar_total_w4

nart_total_w4 ~~ wtar_total_w4

g_w4 ~ 1

blkdes_w4~ 0*1

matreas_w4~ 0*1

spantot_w4~ 0*1

digsym_w4~ 0*1

crtmean_invsq_w4~ 0*1

symsear_w4~ 0*1

ittotal_squared_w4~ 0*1

vpatotal_w4 ~ 0*1

lmtotal_w4~ 0*1

digback_w4 ~ 0*1

‘

fit_A4 <- sem(model = model_A4, model_variables_scaled_completers, missing = "ml.x")

model_A5 <- '

g_w5 =~ blkdes_w5 + matreas_w5 + spantot_w5 + digsym_w5 + symsear_w5 + crtmean_invsq_w5 + ittotal_squared_w5 + vpa_total_w5 + lmtotal_w5 + digback_w5 + vftot_w5 + nart_total_w5 + wtar_total_w5

nart_total_w5 ~~ wtar_total_w5

g_w5 ~ 1

blkdes_w5~ 0*1

matreas_w5~ 0*1

spantot_w5~ 0*1

digsym_w5~ 0*1

crtmean_invsq_w5~ 0*1

symsear_w5~ 0*1

ittotal_squared_w5~ 0*1

vpa_total_w5 ~ 0*1

lmtotal_w5 ~ 0*1

digback_w5 ~ 0*1

‘

fit_A5 <- sem(model = model_A5, data, missing = "ml.x")

R code for Model B (all waves)

library(lavaan)

model_B <- ‘

g_w1 =~ blkdes_w1 + matreas_w1 + spantot_w1 + digsym_w1 + symsear_w1 + crtmean_invsq_w1 + ittotal_squared_w1 + vpatotal_w1 + lmtotal_w1 + digback_w1 + vftot_w1 + nart_w1 + wtar_w1

g_w2 =~ blkdes_w2 + matreas_w2 + spantot_w2 + digsym_w2 + symsear_w2 + crtmean_invsq_w2 + ittotal_squared_w2 + vpatotal_w2 + lmtotal_w2 + digback_w2 + vftot_w2 + nart_w2 + wtar_w2

g_w3 =~ blkdes_w3 + matreas_w3 + spantot_w3 + digsym_w3 + symsear_w3 + crtmean_invsq_w3 + ittotal_squared_w3 + vpatotal_w3 + lmtotal_w3 + digback_w3 + vftot_w3 + nart_total_w3 + wtar_total_w3

g_w4 =~ blkdes_w4 + matreas_w4 + spantot_w4 + digsym_w4 + symsear_w4 + crtmean_invsq_w4 + ittotal_squared_w4 + vpatotal_w4 + lmtotal_w4 + digback_w4 + vftot_w4 + nart_total_w4 + wtar_total_w4

g_w5 =~ blkdes_w5 + matreas_w5 + spantot_w5 + digsym_w5 + symsear_w5 + crtmean_invsq_w5 + ittotal_squared_w5 + vpa_total_w5 + lmtotal_w5 + digback_w5 + vftot_w5 + nart_total_w5 + wtar_total_w5

nart_w1 ~~ wtar_w1

nart_w2 ~~ wtar_w2

nart_total_w3 ~~ wtar_total_w3

nart_total_w4 ~~ wtar_total_w4

nart_total_w5 ~~ wtar_total_w5

# set covariances between gs to 0

g_w1 ~~ 0*g_w2

g_w1 ~~ 0*g_w3

g_w1 ~~ 0*g_w4

g_w1 ~~ 0*g_w5

g_w2 ~~ 0*g_w3

g_w2 ~~ 0*g_w4

g_w2 ~~ 0*g_w5

g_w3 ~~ 0*g_w4

g_w3 ~~ 0*g_w5

g_w4 ~~ 0*g_w5’

fit_B <- sem(model = model_B, data, missing = "ml.x")

R code for Models B1, B2, B3, B4, and B5 (separate models for each wave)

library(lavaan)

model_B1 <- '

g_w1 =~ blkdes_w1 + matreas_w1 + spantot_w1 + digsym_w1 + symsear_w1 + crtmean_invsq_w1 + ittotal_squared_w1 + vpatotal_w1 + lmtotal_w1 + digback_w1 + vftot_w1 + nart_w1 + wtar_w1

nart_w1 ~~ wtar_w1

‘

fit_B1 <- sem(model = model_B1, model_variables_scaled_completers, missing = "ml.x")

model_B2 <- '

g_w2 =~ blkdes_w2 + matreas_w2 + spantot_w2 + digsym_w2 + symsear_w2 + crtmean_invsq_w2 + ittotal_squared_w2 + vpatotal_w2 + lmtotal_w2 + digback_w2 + vftot_w2 + nart_w2 + wtar_w2

nart_w2 ~~ wtar_w2‘

fit_B2 <- sem(model = model_B2,model_variables_scaled_completers, missing = "ml.x")

model_B3 <- '

g_w3 =~ blkdes_w3 + matreas_w3 + spantot_w3 + digsym_w3 + symsear_w3 + crtmean_invsq_w3 + ittotal_squared_w3 + vpatotal_w3 + lmtotal_w3 + digback_w3 + vftot_w3 + nart_total_w3 + wtar_total_w3

nart_total_w3 ~~ wtar_total_w3

‘

fit_B3 <- sem(model = model_B3,model_variables_scaled_completers, missing = "ml.x")

model_B4 <- '

g_w4 =~ blkdes_w4 + matreas_w4 + spantot_w4 + digsym_w4 + symsear_w4 + crtmean_invsq_w4 + ittotal_squared_w4 + vpatotal_w4 + lmtotal_w4 + digback_w4 + vftot_w4 + nart_total_w4 + wtar_total_w4

nart_total_w4 ~~ wtar_total_w4

‘

fit_B4 <- sem(model = model_B4,model_variables_scaled_completers, missing = "ml.x")

model_B5 <- '

g_w5 =~ blkdes_w5 + matreas_w5 + spantot_w5 + digsym_w5 + symsear_w5 + crtmean_invsq_w5 + ittotal_squared_w5 + vpa_total_w5 + lmtotal_w5 + digback_w5 + vftot_w5 + nart_total_w5 + wtar_total_w5

nart_total_w5 ~~ wtar_total_w5

‘

fit_B5 <- sem(model = model_B5, data, missing = "ml.x")

R code for the Hierarchical model (all waves)

hierarchical_model <- '

# specify domains

Vis_w1 =~ blkdes_w1 + matreas_w1 + spantot_w1

Speed_w1 =~ digsym_w1 + symsear_w1 + crtmean_invsq_w1 + ittotal_squared_w1

Mem_w1 =~ vpatotal_w1 + lmtotal_w1 + digback_w1

Cryst_w1 =~ vftot_w1 + nart_w1 + wtar_w1

Vis_w2 =~ blkdes_w2 + matreas_w2 + spantot_w2

Speed_w2 =~ digsym_w2 + symsear_w2 + crtmean_invsq_w2 + ittotal_squared_w2

Mem_w2 =~ vpatotal_w2 + lmtotal_w2 + digback_w2

Cryst_w2 =~ vftot_w2 +nart_w2 + wtar_w2

Vis_w3 =~ blkdes_w3 + matreas_w3 + spantot_w3

Speed_w3 =~ digsym_w3 + symsear_w3 + crtmean_invsq_w3 + ittotal_squared_w3

Mem_w3 =~ vpatotal_w3 + lmtotal_w3 + digback_w3

Cryst_w3 =~vftot_w3 + nart_total_w3 + wtar_total_w3

Vis_w4 =~ blkdes_w4 + matreas_w4 + spantot_w4

Speed_w4 =~ digsym_w4 + symsear_w4 + crtmean_invsq_w4 + ittotal_squared_w4

Mem_w4 =~ vpatotal_w4 + lmtotal_w4 + digback_w4

Cryst_w4 =~ wtar_total_w4 + vftot_w4 + nart_total_w4

Vis_w5 =~ blkdes_w5 + matreas_w5 + spantot_w5

Speed_w5 =~ digsym_w5 + symsear_w5 + crtmean_invsq_w5 + ittotal_squared_w5

Mem_w5 =~ vpa_total_w5 + lmtotal_w5 + digback_w5

Cryst_w5 =~ vftot_w5 + nart_total_w5 + wtar_total_w5

# create g factors

g_w1 =~ Vis_w1 + Speed_w1 + Mem_w1 + Cryst_w1

g_w2 =~ Vis_w2 + Speed_w2 + Mem_w2 + Cryst_w2

g_w3 =~ Vis_w3+ Speed_w3 + Mem_w3 + Cryst_w3

g_w4 =~ Vis_w4 + Speed_w4 + Mem_w4 + Cryst_w4

g_w5 =~ Vis_w5 + Speed_w5 + Mem_w5 + Cryst_w5

# set covariances between gs to 0

g_w1 ~~ 0*g_w2

g_w1 ~~ 0*g_w3

g_w1 ~~ 0*g_w4

g_w1 ~~ 0*g_w5

g_w2 ~~ 0*g_w3

g_w2 ~~ 0*g_w4

g_w2 ~~ 0*g_w5

g_w3 ~~ 0*g_w4

g_w3 ~~ 0*g_w5

g_w4 ~~ 0*g_w5'

fit_hierarchical <- sem(model = hierarchical_model, data, missing = "ml.x")
